# Natural Products from Chinese Medicines with Potential Benefits to Bone Health

**DOI:** 10.3390/molecules21030239

**Published:** 2016-02-27

**Authors:** Chun-Tao Che, Man Sau Wong, Christopher Wai Kei Lam

**Affiliations:** 1 Department of Medicinal Chemistry and Pharmacognosy, College of Pharmacy, The University of Illinois at Chicago, Chicago, IL 60612, USA; 2 Department of Applied Biology and Chemical Technology, The Hong Kong Polytechnic University, Hong Kong, China; man-sau.wong@polyu.edu.hk; 3 State Key Laboratory of Quality Research in Chinese Medicine, Macau Institute for Applied Research in Medicine and Health, Macau University of Science and Technology, Macau, China; wklam@must.edu.mo

**Keywords:** anti-osteoporosis, osteoprotection, bone health, natural product, Chinese medicine

## Abstract

Osteoporosis is a progressive, systemic bone disorder characterized by loss of bone mass and microstructure, leading to reduced bone strength and increased risk of fracture. It is often associated with reduced quality of life and other medical complications. The disease is common in the aging population, particularly among postmenopausal women and patients who receive long-term steroidal therapy. Given the rapid growth of the aging population, increasing life expectancy, the prevalence of bone loss, and financial burden to the healthcare system and individuals, demand for new therapeutic agents and nutritional supplements for the management and promotion of bone health is pressing. With the advent of global interest in complementary and alternative medicine and natural products, Chinese medicine serves as a viable source to offer benefits for the improvement and maintenance of bone health. This review summarizes the scientific information obtained from recent literatures on the chemical ingredients of Chinese medicinal plants that have been reported to possess osteoprotective and related properties in cell-based and/or animal models. Some of these natural products (or their derivatives) may become promising leads for development into dietary supplements or therapeutic drugs.

## 1. Introduction

Osteopenia (low bone density) and osteoporosis (“porous bone”) are progressive metabolic bone disorders occurring in aging populations, especially postmenopausal women and patients who undergo long-term steroid therapy. While postmenopausal women are at greater risk, osteopenia/osteoporosis can strike at any age of both genders. The disease is characterized by thinning of bones, with reduction in bone mass and bone mineral density, as well as micro-architectural deterioration of the bone tissue due to depletion of calcium and bone protein. The clinical manifestation is loss of bone strength, thus making the bones fragile and vulnerable to fractures, which often happens in the hip, spine, and wrist. The disease is often associated with reduced quality of life and other medical complications such as disability and depression. Worldwide, osteoporosis is estimated to affect 200 million women, approximately one-tenth of women aged 60 and one-fifth of women aged 70 [1]. Although the overall prevalence of fragility fractures is higher in women, osteoporosis is a significant health issue in men [2]. The U.S. National Osteoporosis Foundation projected that by 2020, fourteen million Americans over the age of 50 are expected to have osteoporosis and another 47 million to have low bone mass, accounting for 55% of the population 50 years of age and older [3].

In comprehensive bone health management plans, both pharmacologic therapy and non-pharmacologic measures (such as balanced diet, adequate calcium and vitamin D intake, exercise and fall prevention) are usually included [4,5,6]. Currently there are less than ten FDA-approved drugs for osteoporosis prevention and treatment. They fall into two classes, the anti-resorptive and the anabolic drugs. Anti-resorptive drugs include bisphosphonates (alendronate, ibandronate, risedronate, and zoledronic acid), calcitonin, denosumab (an inhibitor of receptor activator of nuclear factor-κB ligand [RANKL]), and raloxifene (a selective estrogen receptor modulator). These compounds slow down the process of bone loss. On the other hand, anabolic drug such as the recombinant form of parathyroid hormone (teriparatide) enhances new bone formation [7,8,9]. Nevertheless, the efficacy of these drugs varies in patients and the long-term safety has posted some concerns; for example, potentially serious adverse effects of bisphosphonate therapy have been reported [10,11,12,13,14,15]. Only a small number of investigational drugs are currently in the pipeline of development [16,17], and new targets such as c-Src kinase, cathepsin K, and chloride channel are being investigated [18,19,20].

Estrogen (with or without progesterone) has been used in hormone replacement therapy. It is effective to increase bone density and reduce the risk of fracture. Nevertheless, the use of estrogens has been restrained due to concerns of risk of cancer (such as breast, endometrial and ovarian cancers), heart attack and stroke [21]. Dietary supplementation of calcium and vitamin D is often included as part of the treatment plan, yet calcium and vitamin D alone or in combination are ineffective in reducing fractures in the absence of pharmacologic agents [22,23].

Given the rapid growth of the aging population, increasing life expectancy, the prevalence of bone loss, and financial burden to the healthcare system and individuals, demand for new therapeutic agents and nutritional supplements for the management and promotion of bone health is pressing. With the advent of general interest in alternative medicine and natural products, Chinese medicine can serve as a viable source to offer benefits for the improvement and maintenance of bone health. This review intends to highlight scientific information on naturally-occurring chemical compounds derived from Chinese medicinal plants which have been documented to possess protective properties against osteoporosis or osteonecrosis. Focus has been put on worldwide literature available in the last ten years. Due to the vast volume of literature information readily obtainable for the soybean phytoestrogens (such as daidzein, genistein and equol) [24,25,26,27,28,29,30], this class of compounds is not included in the present review. In addition, this review covers only natural molecules derived from Chinese medicinal plants; and it does not include herbal extracts and medicinal formulas from which the active compounds are unidentified. Readers are referred to a number of recent review articles on the topics of medicinal herbs and/or Chinese medicines for bone disorders and for maintenance of bone health [31,32,33,34,35,36,37,38,39,40,41,42].

## 2. Indicators of Bone Health

Bone is a living and growing tissue that constantly forms and breaks down. The “remodeling” process is a continuous renewal and replacement of bone tissues, in which there are two distinct phases: bone formation and bone resorption (breakdown or removal). Two kinds of specialized cells are involved in bone remodeling. During bone formation, the *osteoblasts* (bone-forming cells) fill up the bone cavities with new tissues; and during resorption, the *osteoclasts* dissolve bone tissues. Under normal and healthy condition, bone resorption and formation take place in a dynamic and balanced manner so that the old tissues are constantly replaced by new tissues. However, under an imbalanced situation in which the process of bone resorption is faster than that of bone formation, bone tissues will be lost and osteoporosis results (clinically manifested by lowered bone mineral density).

Since bone is the major storage site for calcium in the body, calcium is critically important to bone health, with calcium phosphate and mineralized collagen being the structural supporting materials of the bone tissue. When the blood calcium level is low, a series of physiological responses (such as induction of parathyroid hormone that triggers the release of bone calcium, increase of intestinal calcium absorption and increase of renal calcium reabsorption) will help to maintain calcium homeostasis. Conventionally, the serum and urine calcium levels are used as indicators of mineral balance in the body. In particular, excessive urinary calcium excretion is associated with bone loss and osteoporosis.

For bone health assessment and evaluation of therapeutic responses, a number of indicators have become available for clinical tests [43]. Many of these indicators are also applicable to experimental studies using *in vitro* (cell-based) assays or *in vivo* (animal) models. Indicators of bone remodeling include the measurements of bone mineral density, bone microarchitecture, and biochemical markers.

Bone mass can be estimated by two approaches: (a) measurement of the amount of calcified tissue in bone tissue, *i.e.*, bone mineral density (BMD); and (b) measurement of bone quality such as trabecular microarchitecture and fragility [44]. As the prime indicator of bone strength, BMD has become the standard for diagnosis of osteoporosis as recommended by the World Health Organization [45]. While BMD is a quantitative assessment of bone health, it does not provide information on bone quality. On the other hand, bone microarchitecture is related to the mechanical strength, and deterioration of bone architecture results in bone loss, as shown by observations such as decreased number of trabeculae, increased inter-trabecular distances, loose connectivity of the trabecular meshwork, reduction of cortical bone thickness and increased porosity [46]. Apart from BMD measurement and bone microstructure assessment, bone strength and fragility tests are often employed in animal models to test for drug effects.

In recent years cellular components of the bone tissue have also been widely used as biomarkers to measure and monitor bone turnover and bone loss [47,48,49,50], and new markers are being developed [51]. The markers reflect the metabolic activity of osteoblasts or osteoclasts and are measurable in blood or urine in order to provide a quantitative estimate of the status of bone remodeling. Information on bone remodeling status is an early indicator of pathological changes or the risk of some bone diseases. These biomarkers are useful not only for clinical assessments as monitors of osteoporosis and predictors of fracture, but also for the evaluation of therapeutic responses [49]. By measuring the concentration/activity of biomarkers, it is possible to obtain information about therapeutic response faster than by measuring bone mass. Significant changes in biochemical markers may be readily observed after one to three months of effective therapy. The most useful biomarkers are either enzymes secreted by osteoclasts or osteoblasts, or proteins or their fragments produced by osteoblasts during bone formation or released by degradation of the collagen matrix of bone during resorption. In general, these biomarkers fall into two categories, *i.e.*, bone formation markers and bone resorption markers, depending on which phase of bone remodeling they reflect (Table 1).

### 2.1. Bone Formation Markers

Markers of bone formation are osteoblastic enzymes or by-products of active osteoblasts expressed during their developmental process. The most common biomarkers include alkaline phosphatase, osteocalcin, and the carboxy- and amino-terminal propeptides of type 1 collagen.

*Alkaline phosphatase* is derived from various tissues including the liver and bone. In the bone, the enzyme is present in osteoblast plasma membranes and plays an important role in osteoid formation and mineralization [52]. Total alkaline phosphatase in serum is a widely used non-specific marker of bone formation, but detection of the bone-specific isoenzyme (the bone-specific alkaline phosphatase) is increasingly preferred due to its higher specificity [53].

*Osteocalcin* is the most abundant non-collagen protein in the bone matrix. It is produced by mature osteoblasts during bone formation and acts in the bone matrix to regulate mineralization [56]. Osteocalcin is released into the circulation from the matrix during bone resorption and, therefore, is considered a marker of bone turnover rather than a specific marker of bone formation, despite its serum levels correlate well with the osteoblast activity and bone mineral density [57].

Procollagen I is a precursor of collagen. When the collagen is made and deposited to form the bone matrix, both the C- and N-terminals of procollagen I are removed by specific proteases. The *procollagen 1 C terminal extension peptide* (P1CP) and *procollagen 1 N terminal extension peptide* (P1NP) are thus specific products of proliferating osteoblasts and fibroblasts and they can serve as markers of bone formation. P1NP seems to have several advantages over P1CP such as lower individual variability and higher stability. It became a marker of choice recommended by professional bodies such as the International Osteoporosis Foundation (IOF), International Federation of Clinical Chemistry and Laboratory Medicine (IFCC), and National Bone Health Alliance (NBHA) [59,63].

### 2.2. Bone Resorption Markers

The matrix of bone is largely constituted by type I collagen. During the degradation process, breakdown products are released and pass into the blood stream and/or urine. Most biochemical markers of bone resorption are based on collagen breakdown products such as deoxypyridinoline or the collagen cross-links and telopeptides. Two other types of markers include osteoclast-specific enzymes such as tartrate-resistant acid phosphatase (TRAP) and cathepsin K, and osteocyte activity markers such as receptor activator of nuclear factor κB ligand (RANKL) and osteoprotegerin (OPG).

*Tartrate-resistant alkaline phosphatase* (TRAP) is characteristic of osteoclasts, and it is expressed and secreted primarily by osteoclasts during active bone resorption. It is, however, also produced by inflammatory macrophages and dendritic cells. The serum level is a useful biochemical marker to assess osteoclast activity. More recently, the TRAP 5b isoform was shown to be osteoclast-specific and serves as a better indicator of bone resorption and osteoclast number [54].

*Cathepsin K* is a cysteine protease enzyme present in actively resorbing osteoclasts. It is able to cleave the telopeptide region of type 1 collagen. The serum level of cathepsin K reflects the number of osteoclasts and serves as a specific biomarker of osteoclast activity [55].

*Hydroxyproline* is a component of bone collagen. During the process of bone degradation, hydroxyproline is liberated from collagen and excreted in the urine where it can be detected as free or bound forms. Hydroxyproline is considered a nonspecific marker of bone turnover, since it is also a degradative product of collagens from tissues other than bone, and it can also come from diet [57].

The pyridinium compounds, *pyridinoline* and *deoxypyridinoline*, are formed during the extracellular maturation of fibrillar collagens and are released upon the degradation of mature collagens. The measurement of pyridinoline and deoxypyridinoline is not influenced by degradation of newly synthesized collagens and is independent of dietary sources [58].

Assays are available based on specific antibodies raised against collagen peptides containing cross-links. Thus, assays for *Carboxy-terminal cross-linked telopeptide of type 1 collagen* (CTX) and *amino-terminal cross-linked telopeptide of type 1 collagen* (NTX) are employed as indicators of bone resorption. The CTX assay has been recommended by professional bodies such as IOF, IFCC, and NBHA [59,63].

*Osteopontin* (OPN), also known as bone sialoprotein (BSP), is a phosphorylated glycoprotein present in the non-collagenous matrix of bone. It plays the role of anchoring osteoclasts to the mineral matrix. Serum OPN thus reflects the process of osteoclast activity and bone resorption [60].

*Receptor activator of nuclear factor Kappa B ligand* (RANKL), is a pro-osteoclastogenic cytokine produced by the osteoblasts. Binding to the RANK on osteoclasts leads to the activation of downstream signaling pathways such as MAPKs, NF-κB and NFATc1 [62,64,65]. As a result, the RANKL pathway is associated with the stimulation of osteoclast differentiation. The RANKL level serves as a marker for bone loss [61].

*Osteoprotegerin* (OPG) is secreted by the osteoblasts and it binds to RANKL to inhibit the differentiation of the osteoclast precursor into mature osteoclast, thus suppressing osteoclastic growth [62,64,65]. Measurement of the OPG/RANKL ratio has been established as an indicator of osteoclastogenesis.

## 3. Natural Products with Osteoprotective and Related Activities

### 3.1. Carthami Flos (Flower of Carthamus tinctorius L.)

Carthami Flos, known as safflower or “Hong-Hua”, is used in Chinese medicine to promote blood circulation, treat traumatic damages and ease muscle pains. In Korean traditional medicine, the seeds of this plant are used to promote bone formation and prevent osteoporosis.

**Figure 1:**
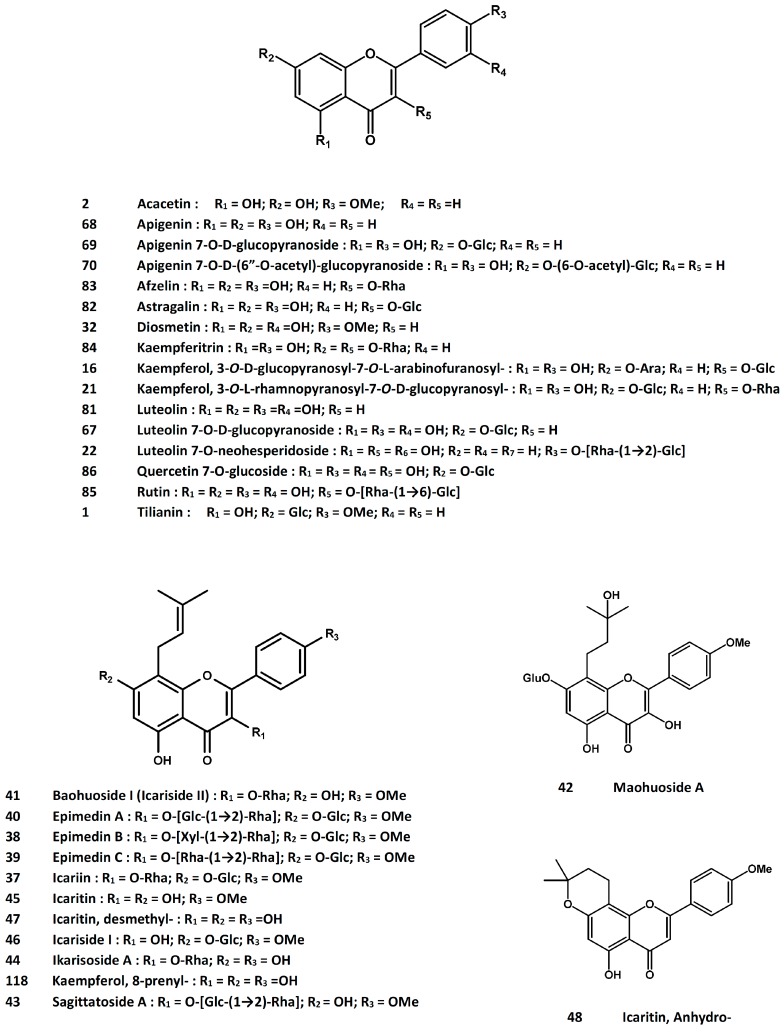
Flavones and Flavonols Reported to Possess Osteoprotective Property.

**Figure 2:**
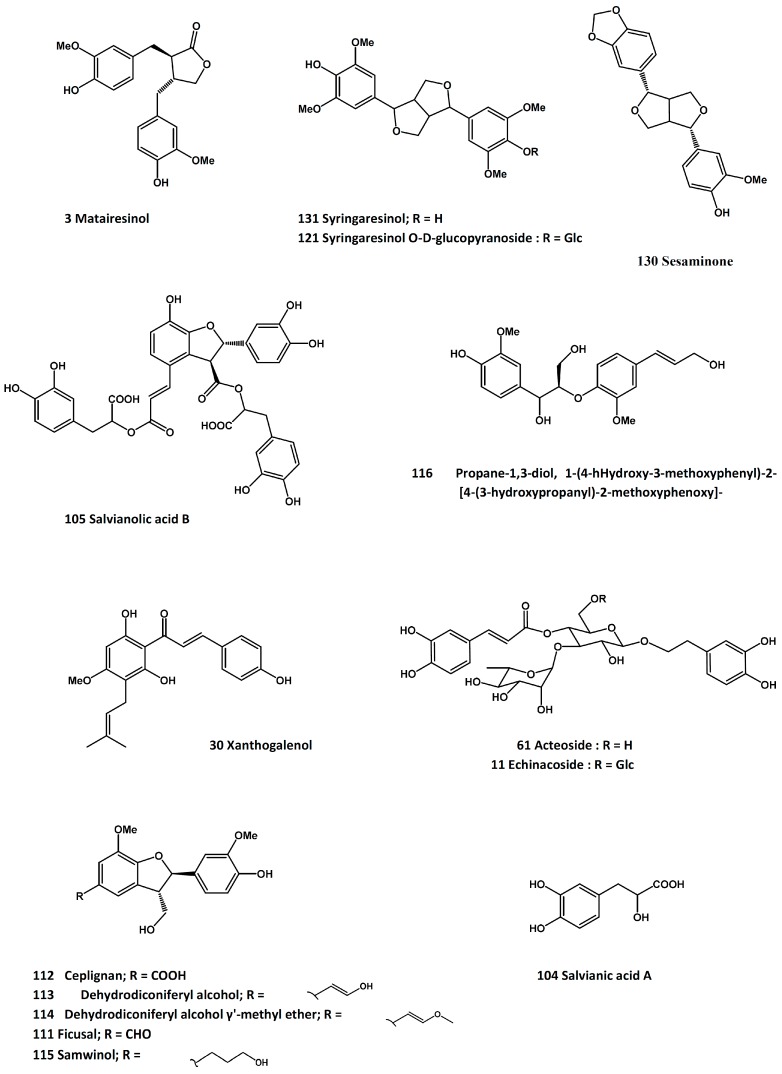
Phenylpropanol Derivatives and Lignans Reported to Possess Osteoprotective Property.

The defatted seeds of *C. tinctorius* have been shown to protect ovariectomized rats from trabecular bone loss and the phenolic compounds-rich fraction stimulated the growth of osteoblast-like ROS 17/2.8 cells [66]. The safflower seed oil also displayed protective effect in ovariectomized rats by increasing the serum levels of insulin-like growth factors I and II, insulin-like growth factor binding protein-3, and alkaline phosphatase activity [67]. In 3-week-old rats, feeding with a methanol extract of safflower led to increased serum markers such as osteocalcin, alkaline phosphatase activity, and insulin-like factor I level, as well as the growth parameters such as length of femur and tibia [68]. Both the crude extract and the aqueous extracts promoted osteoblast differentiation in MC3T3-E1 cells [69]. The osteoprotective property of safflower was also associated with the inhibition of bone resorption. The response has been related to inhibition of the Src family kinases [70] and inhibition of osteoclast differentiation, as evidenced by the suppression of tartrate-resistant acid phosphatase (TRAP)-positive multinucleated cells, gene expressions of the nuclear factor of activated T-cells (NFATc1), receptor activator of nuclear factor-κB ligand (RANKL)-mediated intracellular reactive oxygen species (ROS) generation, p38 mitogen-activated protein kinase and IκB-β kinase signaling activities; RANKL-induced actin ring formation was also suppressed [71]. The active ingredients of safflower seeds have been proposed to be the polyphenolic compounds such as tilianin (**1**, Figure 1), acacetin (**2**, Figure 1), matairesinol (**3**, Figure 2), and their derivatives [72].

### 3.2. Cimicifugae Rhizoma (Rhizome of Actaea heracleifolia (Kom.) J. Compton [syn. Cimicifuga heracleifolia Kom.], A. dahurica (Turcz. ex Fisch. and C.A. Mey.) Franch. [syn. C. dahurica (Turcz.) Maxim.], or A. foetida L. [syn. C. foetida L.])

Cimicifugae Rhizoma, known as “Sheng-Ma” in Chinese medicine, is renowned for its heat-repelling and detoxifying effects. Clinically it is used in combination with other herbs to treat symptoms caused by excessive heat in the body, such as gum infection, mouth sores, sore throat, fever, rash, and skin diseases. More recent applications of this plant drug include the treatment of hemorrhoids, Parkinson’s syndrome, and menopausal symptoms.

**Figure 3:**
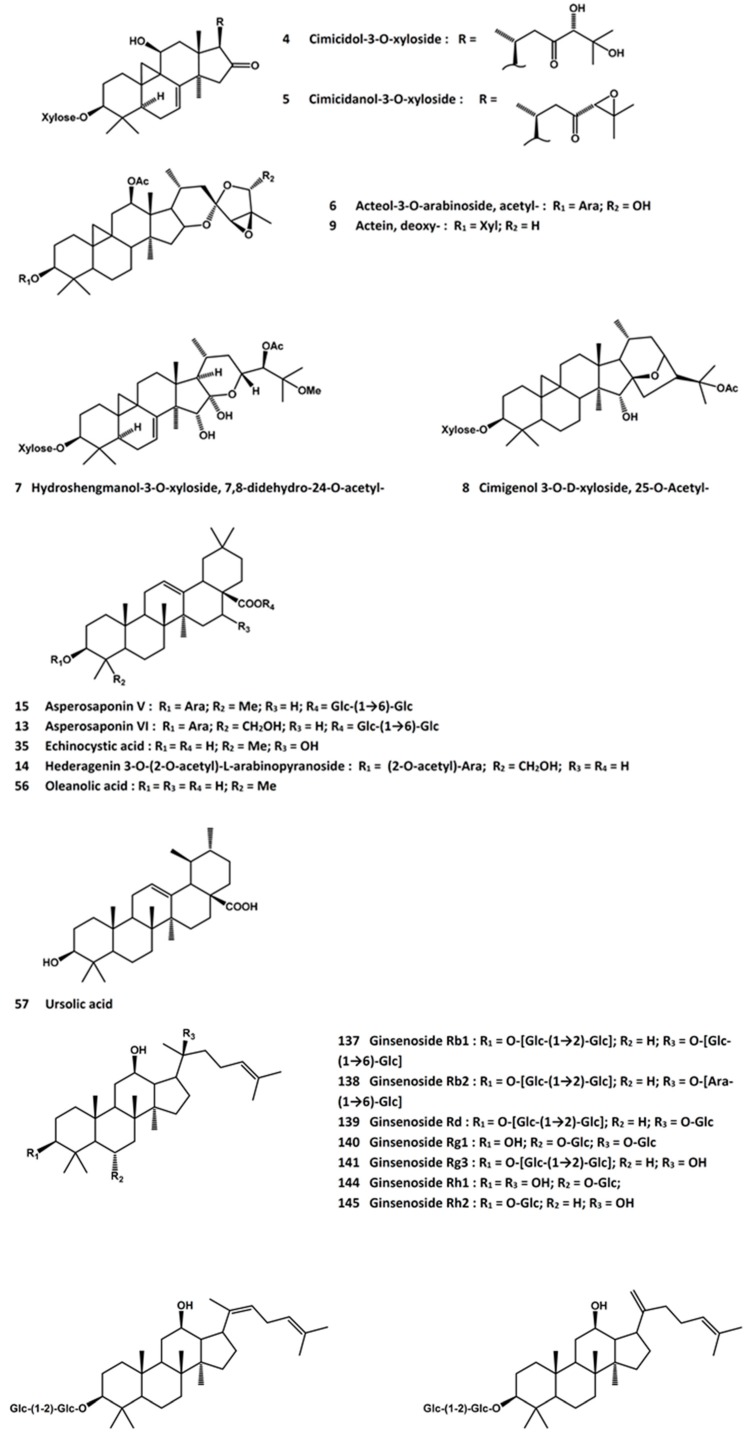
Triterpenoids reported to possess osteoprotective property.

After feeding the ethyl acetate-soluble extracts of either *A. heracleifolia* or *A. foetida* to ovariectomized rats for 42 days, the serum calcium levels were decreased whereas the bone mineral density was increased in the lumbar spine [73]. Four triterpene compounds isolated from the extracts, namely, cimicidol-3-*O*-β-xyloside (**4**, Figure 3), cimicidanol-3-*O*-β-xyloside (**5**, Figure 3), acetylacteol-3-*O*-arabinoside (**6**, Figure 3), and 7,8-didehydro-24-*O*-acetyl-hydroshengmanol-3-*O*-β-xyloside (**7**, Figure 3), were shown to decrease the circulating calcium levels in ovariectomized rats [73]. In a subsequent study, the former three triterpenes were demonstrated to suppress the formation of osteoclast-like cells and their resorbing activity [74]. More recent results showed that an extract of *A. heracleifolia* prevented the ovariectomy-induced elevation of serum alkaline phosphatase level; it was able to preserve trabecular bone mass, bone volume, trabecular number, trabecular thickness, structure model index as well as bone mineral density of the proximal tibia metaphysis and distal femur metaphysis in ovariectomized mice [75].

*Actaea racemosa* is a related species native to North America. The roots and rhizomes, known as black cohosh, in widely used for the management of symptoms associated with menopause. It enhanced osteoblastic differentiation and increased the osteoprogeterin (OPG)/RANKL ratio in human osteoblasts [76]. A standardized black cohosh preparation was found to prevent bone density loss in the distal end of the femur and preserve the trabecular bone structure in the lumbar vertebra and femur in ovariectomized rats [77]. A triterpene saponin fraction of the plant extract was claimed to be responsible for the osteoprotective effect [78]. 25-*O*-Acetylcimigenol 3-*O*-β-xylopyranoside (**8**, Figure 3) was able to block *in vitro* osteoclastogenesis induced by RANKL or tumor necrosis factor (TNF)-α, and it attenuated TNF-α-induced bone loss in mice [79]. From black cohosh, deoxyactein (**9**, Figure 3) has also been isolated and demonstrated to promote cell growth, alkaline phosphatase activity, collagen content, and mineralization of MC3T3-E1 cells. In the presence of the reactive oxygen species (ROS) generator antimycin A, deoxyactein was able to suppress the production of ROS and osteoclast differentiation factors such as TNF-α, interleukin (IL)-6, and RANKL [80].

### 3.3. Cistanches Herba [Stem of Cistanche deserticola Y.C. Ma or C. tubulosa (Forssk.) Beck]

Cistanches Herba, known in Chinese medicine as “Rou-Cong-Rong”, is a kidney- and Yang-tonifying drug that can invigorate the body essence and blood. Common uses and indications include general physical weakness, fatigue, low back pain and joint weakness.

**Figure 4:**
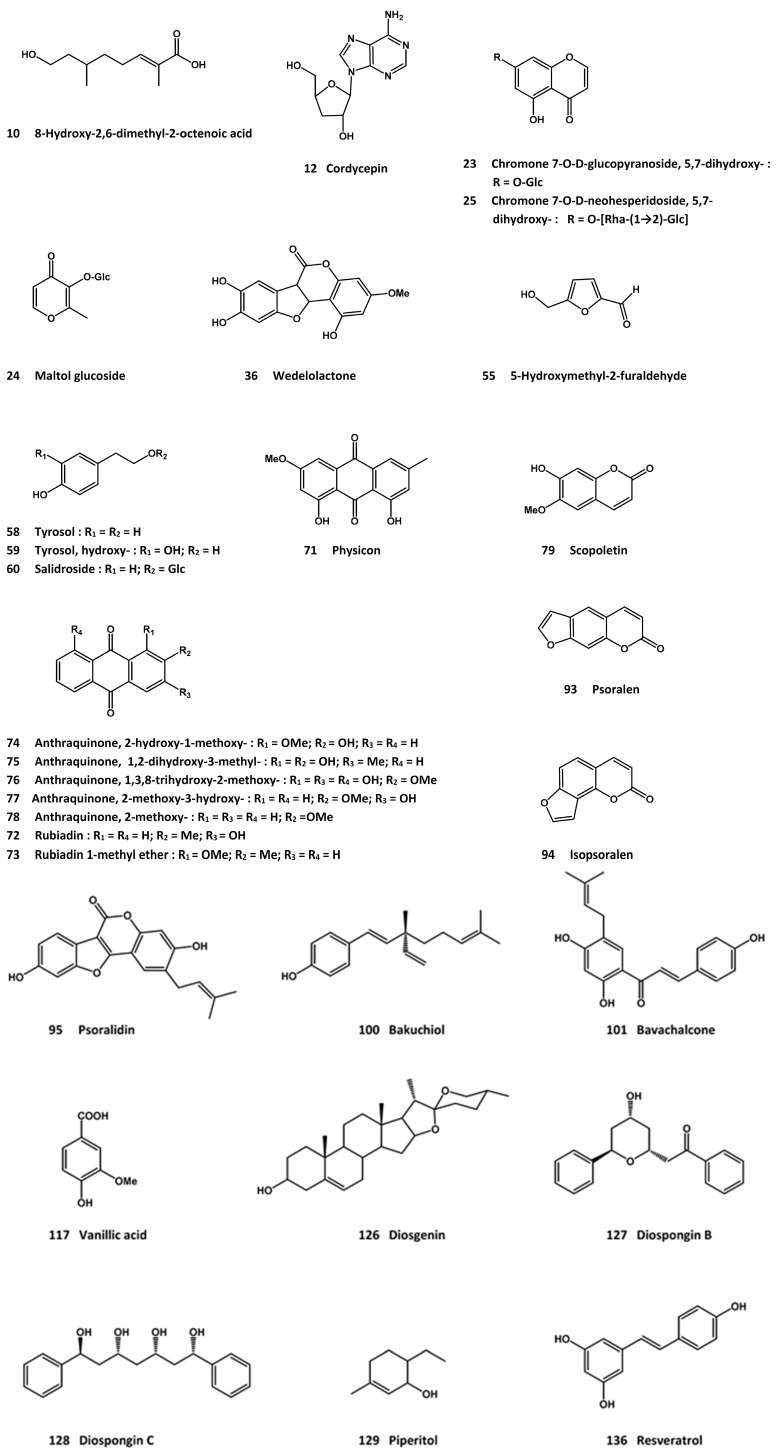
Miscellaneous compound types reported to possess osteoprotective property.

In an early study, a monoterpene, 8-hydroxy-2,6-dimethyl-2-octenoic acid (**10**, Figure 4), was identified as an anti-osteoporotic compound from a related plant species, *C. salsa* [81]. More recently, the plant drug has been revisited for its anti-osteoporotic potential. Thus, an aqueous *Cistanches* extract dose-dependently enhanced bone mineral density and bone mineral content, as well as improved bone biomechanical indices such as maximum load, displacement at maximum load, stress at maximum load, load at auto-break, displacement at auto-break, and stress at auto-break, in ovariectomized rats [82]. The extract was also reported to protect against ovariectomy-induced bone degeneration, by regulating bone metabolic genes such as Smad1, Smad5, TGF-β1, and TIEG1 [83]. An extract of *C. deserticola* was reported to exhibit promotional activity on osteoblast differentiation as evidenced by increased alkaline phosphatase activity and mRNA expressions of bone morphogenetic protein (BMP)-2 and osteopontin (OPN) [84].

Echinacoside (**11**, Figure 2), a phenylpropanoid glycoside also present in *Echinacea* spp., has been isolated from *C. tubulosa* and found to cause significant increases in MC3T3-E1 cell proliferation, alkaline phosphatase activity, collagen 1 secretion, osteocalcin levels, and mineralization [85]. The authors concluded that osteoprotegerin (OPG) and RANKL may be involved in the anti-osteoporotic process of echinacoside [85]. In a follow-up *in vivo* study, the research team demonstrated the counteracting effects of echinacoside against ovariectomy-induced damages, leading to improvements of bone mineral density, bone biomechanical properties, microarchitecture, histomorphology, and uterus immunohistochemistry [86,87].

### 3.4. Cordyceps [Cordyceps sinensis (Berk.) Sacc.]

*Cordyceps sinensis*, “Dong-Chong-Xia-Cao” in Chinese medicine, is a parasitic fungus which infects caterpillar larvae and eventually replaces the host tissue. Traditionally, the crude drug of cordyceps is comprised of the body of caterpillar larvae being filled up inside by fungal filaments. In current dietary supplement markets, fermentation products of Cordyceps fungal filaments are also available. In addition to *C. sinensis*, few other related species, such as *C. militaris* (L.) Fr., have been used as alternative natural sources. Cordyceps is highly regarded as a kidney- and lung-tonifying agent, particular useful for improving general physical weakness, easing respiratory discomforts, and relieving low back pain.

In an early study, the water extract of *C. sinensis* was found to inhibit the RANKL-induced osteoclast differentiation and suppress the mRNA expressions of osteoclast-related genes such as calcitonin receptor, cathepsin K, matrix metalloprotease 9, and nuclear factor of activated T cells c1 (NFATc1) [88]. When ovariectomized rats were treated with cordyceps together with strontium, beneficial effects were obvious, such as improved bone mechanical strength and bone mineral content, and decreased urinary calcium excretion [89]. A follow-up study indicated that the combination treatment led to a decrease in alkaline phosphatase activity, TRAP activity, cross-linked telopeptides of collagen type I level, and interferon-γ level [90]. Using a rat model of disuse-induced bone loss and deterioration of trabecular microarchitecture, researchers have shown that an extract of *C. sinensis* exerted positive effects on improving the mechanical strength, bone mineral density and bone mineral content [91]. The extract also decreased bone turnover markers and increased osteocalcin levels. Micro-computed tomography (micro-CT) analysis revealed a preventive action on the reduction of the bone volume fraction connectivity density, and trabeculae number and thickness in the hind-limb suspended animals [91].

A peptide called cordymin isolated from *C. sinensis* was demonstrated to play a protective role in diabetic osteopenia in alloxan-induced diabetic rats [92]. On the other hand, an isoflavone mixture obtained from *C. sinensis* was reported to exert beneficial effects on osteoporosis in ovariectomized rats, the effect being attributed to the decrease of alkaline phosphatase activity, TRAP activity, C-terminal crosslinked telopetides of collagen type 1 (CTX), and interferon-γ levels [93].

Cordycepin (**12**, Figure 4) is a nucleoside analog (3’-deoxyadenosine) found in *C. militaris*. It has been shown to inhibit RANKL-induced osteoclast differentiation, and down-regulate the mRNA expressions of osteoclastogenesis-related genes such as tartrate-resistant alkaline phosphatase (TRAP), cathepsin K, matrix metalloproteinase (MMP)-9 and NFATc1 [94]. In a mouse model of lipopolysaccharide-mediated bone loss, cordycepin suppressed the inflammatory bone loss based on micro-CT analysis of the femurs [94]. It also acted as an anti-inflammatory agent by down-regulating proinflammatory cytokines such as IL-1β and TNF-α in a magnesium silicate-induced inflammatory osteoporotic model [95]. In an attempt to understand the mechanisms involved in inflammatory cytokine-induced osteogenesis, cordycepin was found to protect against TNF-α-induced inhibition of osteogenic differentiation of the human adipose-derived mesenchymal stem cells; it restored the mRNA levels of Runt-related transcription factor 2 (Runx2) and osterix (Osx) [96], both of which are transcription factors associated with osteoblast differentiation. A recent study demonstrated that an 8-week cordycepin treatment in ovariectomized rats resulted in decreased levels of alkaline phosphatase activity, TRAP, and CTX, with a concomitant increase of osteocalcin level. Histological examination showed that cordycepin treatment was able to prevent bone loss caused by estrogen deficiency [97].

### 3.5. Dipsaci Radix (Root of Dipsacus asper Wall. ex C.B. Clarke [syn. D. asperoides C.Y. Cheng and T.M. Ai] or D. japonicas Miq.)

Dipsaci Radix is a tonic drug for the liver and kidney; it also promotes blood circulation and alleviates pain. The Chinese drug name “Xu-Duan” implies its therapeutic effects on promoting tendon and bone regeneration. It is therefore often used for bone and related disorders, such as muscle injuries, bone fracture, lower back pain and knee weakness.

An animal study has demonstrated that feeding Dipsaci Radix to normal mice resulted in an increase in bone/tissue volume ratio as well as an increase in trabecular bone number, implying an elevation of bone density and altered bone histomorphology [98]. In another study, when an extract was fed to ovariectomized rats starting at four weeks after removal of the ovary and lasted for 16 weeks, loss of bone mass was prevented, which was supported by decreased levels of bone turnover markers such as serum alkaline phosphatase, osteocalcin, and urinary deoxypyridinoline. Treatment with the plant extract also enhanced bone biomechanical strength and slowed down the deterioration of trabecular microarchitecture [99]. More recently, Dipsaci Radix was reported to prevent the loss of bone mass in a hind-limb unloading rat model; it exhibited beneficial effects on mechanical strength, bone mineral density, bone mineral contents, bone turnover markers, and the changes in urinary calcium and phosphorus excretion [100].

The osteoprotective activity of Dipsaci Radix has been associated with its saponin constituents. Thus, the total saponin fraction of the extract induced cell maturation and differentiation in MC3T3-E1 and primary osteoblastic cells through an enhancement of BMP-2 formation [101]. Further mechanistic studies revealed that the cell differentiation activity was associated with an increase in the expressions of phosphorylated-Smad1/5/8, p-Erk1/2, p-p38 and Runx2. Blocking the BMP-2 expression by noggin significantly reduced the levels of osteoblastic differentiation [102]. A saponin constituent, asperosaponin VI (**13**, Figure 3), was demonstrated to be an active ingredient [103]. In another report, a dichloromethane fraction of Dipsaci Radix was shown to enhance the osteoblastic differentiation of alveolar bone marrow-derived mesenchymal stem cells; and a triterpene glycoside, hederagenin 3-*O*-(2-*O*-acetyl)-α-l-arabinopyranoside (**14**, Figure 3), was found to be active in increasing the alkaline phosphatase activity and protein expressions of sialoprotein and osteocalcin in the differentiated cells [104]. In addition, in a zebrafish model for screening anti-osteoporotic activity, the column fractions of Dipsacus Radix [105], as well as asperosaponins V (**15**, Figure 3) and VI [106], displayed protective effects.

### 3.6. Drynariae Rhizoma [Rhizome of Drynaria fortunei (Kunze ex Mett.) J. Sm. (syn. D. roosii Nakaike)]

Drynaria Rhizoma, literally meaning “healing broken bone” in Chinese language, is a renowned liver- and kidney-tonifying herb for use in bone fracture, traumatic damages, low back pain, and muscle weakness.

*In vitro* studies have demonstrated an extract of *D. fortunei* being able to suppress osteoclast activities such as down regulation of osteopontin and osteonectin mRNA expressions [107,108]. The bone protective effect seemed to be associated with interrupting the trafficking of pro-cathepsin K in osteoclasts [109]. More recently, the total flavonoids from Drynariae Rhizoma were reported to suppress the expression of cathepsin K [110,111]. In animal studies, the herbal drug increased bone density in normal mice [112] and improved cancellated bone ultra-microstructure as well as proline hydroxylation level in ovariectomized rats [113]. Improved biomechanical and histomorphometric conditions in ovariectomized rats were also reported [114].

**Figure 5:**
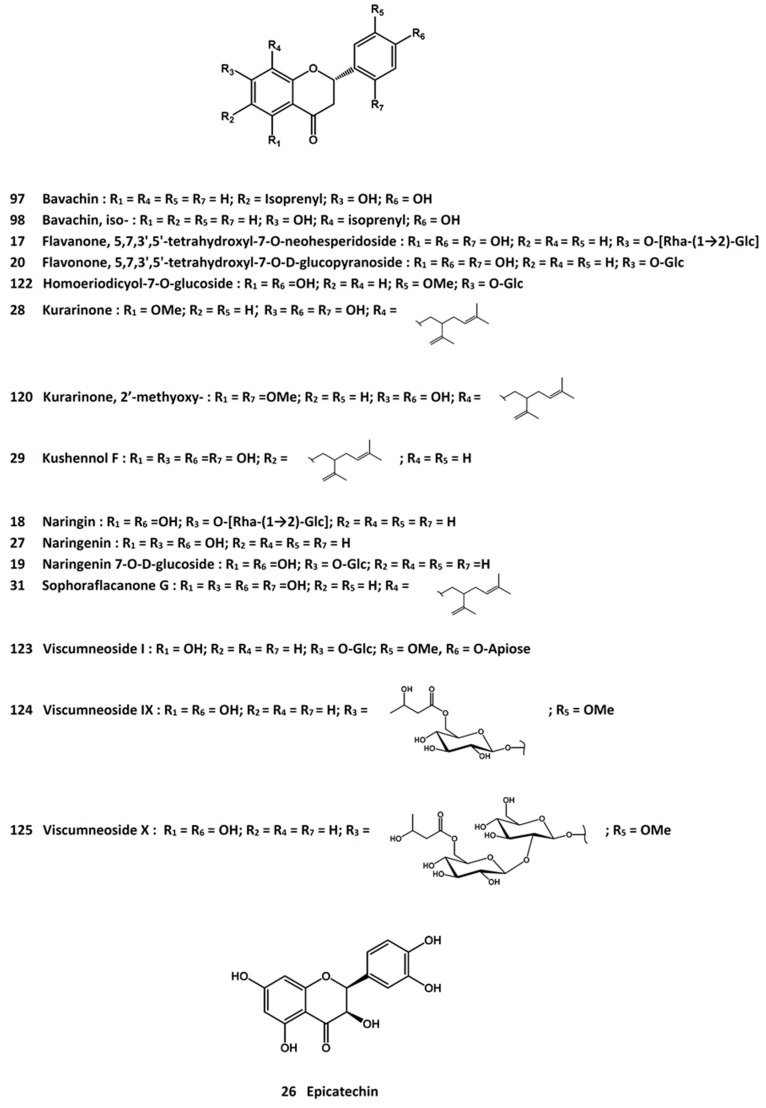
Flavanones Reported to Possess Osteoprotective Property.

In an attempt to identify the active principles from *D. fortunei*, eleven flavonoids were obtained by bioactivity-guided isolation procedures and all were found to display proliferative activity in UMR-106 cells [115]. These active compounds were, namely, 3-*O*-β-d-glucopyranosyl-7-*O*-α-l-arabinofuranosyl-kaempferol (**16**, Figure 1), (2*S*)-5,7,3’,5’-tetrahydroxylflavanone-7-*O*-neohesperidoside (**17**, Figure 5), (2*R*)-naringin (**18**, Figure 5), (*S*)-naringenin 7-*O*-β-d-glucoside (**19**, Figure 5), (2*S*)-5,7,3’,5’-tetrahydroxylflavonone 7-*O*-β-d-glucopyranoside (**20**, Figure 5), 3-*O*-α-l-rhamnosyl-7-*O*-β-“d-glucopyranosyl-kaempferol (**21**, Figure 1), luteolin 7-*O*-β-d-neohesperidoside (**22**, Figure 1), 5,7-dihydroxychromone 7-*O*-β-d-glucopyranoside (**23**, Figure 4), maltol glucoside (**24**, Figure 4), dihydroxychromone 7-*O*-β-d-neohesperidoside (**25**, Figure 4), and (−)-epicatechin (**26**, Figure 5) [115]. In another study, five flavonoid derivatives, *i.e.*, naringenin (**27**, Figure 5), kurarinone (**28**, Figure 5), kushennol F (**29**, Figure 5), xanthogalenol (**30**, Figure 2), and sophoraflavanone G (**31**, Figure 5), demonstrated promoting activity in the differentiation and mineralization of UMR-106 cells, likely to achieve through the activation of the estrogen receptor signaling pathway [116]. On the other hand, naringin, (2*S*)-5,7,3’,5’-tetrahydroxylflavonone 7-*O*-neohesperidoside (**17**, Figure 5), and 5,7-dihydroxy-chromone 7-*O*-β-d-neohesperidoside (**25**, Figure 4) were shown to stimulate UMR-106 cell proliferation and alkaline phosphatase activity; they enhanced the ratio of osteoprotegrin and RANKL mRNA expression [117]. Naringin [118] and total flavonoids [119] were also able to promote the proliferation and osteogenic differentiation of human bone mesenchymal stem cells. Using the MC3T3-E1 cells, both Drynariae Rhizoma extracts and naringin promoted cell proliferation and differentiation [120]. They reduced bone resorption in a rat model of alveolar bone resorption as well [120]. Increased trabecular-rich bone mineral density at distal femur and lumbar spine in ovariectomized mice was reported for the total flavonoids; the activity was associated with the stimulation of estrogen receptors α and β [121] and ostocalcin-involved endochondral ossification [114]. The total flavonoid fraction has also been shown to induce osteoblastic differentiation from bone marrow mesenchymal stem cells, with increased mRNA expressions of the Wnt/β-catenin signaling pathway related factors such as β-catenin, lymphoid enhancer-binding factor (LEF)-1 and cycline D [122]. In another study, D. fortunei extract was found to promote osteoblast maturation through regulating bone differentiation-related gene expressions such as those of osteoprogenitor proliferation-related insulin-like growth factor (IGF)-1, BMP-2 and BMP-6 [123].

All in all, naringin (**18**, Figure 5) (which is also rich in citrus fruits) appeared to be the major osteo-active ingredient of Drynaria furtunei [124]. Thus, it was shown to promote *in vitro* osteoblastic proliferation and differentiation in stem cells and osteoblast cell lines [117,118,125,126,127,128,129,130,131,132,133], and the activity might involve an increase in BMP-2 expression via the phosphoinositide 3-kinase (PI3K), Akt, c-Fos/c-Jun and AP-1-dependent signaling pathway [126], the BMP-4 and Wnt-β-catenin pathway [131], and/or an upregulation of microRNA-20a and down-regulation of the peroxisome proliferator-activated receptor γ (PPARγ) [133]. In a study of the potential application of naringin for regenerative treatment of inflammation-induced bone injury, co-administration of TNF-α and naringin to bone marrow mesenchymal stem cells resulted in a protection of the TNF-α-induced damages such as cell death, suppressed alkaline phosphatase activity and expressions of Runx2 and Osx [134]. The protective activity was related to inhibition of the NF-κB pathway [134] On the other hand, the anti-osteoclastogenic property of naringin was demonstrated in both mouse and rabbit osteoblasts [135]. It inhibited osteoclast formation and bone resorption in RAW 264.7 cells [136], possibly as a result of an inhibition of RANKL-induced NF-κB and phosphorylation of ERK [137]. Apoptosis of RAW 264.7 cells has also been observed [138].

Animal studies further indicated the osteoprotective property of naringin. In mice and rats, it reversed the ovariectomy-induced osteoporosis as demonstrated by increased bone mineral density, bone volume, trabecular thickness, polar stress-train index, as well as the biomechanical strength (ultimate load and energy for breaking) [128,129,138]. Similarly, in a rat model of retinoic acid-induced osteoporosis, treatment with naringin resulted in higher femur bone mineral density, improved bone weight coefficient, bone size, and bone ash [127,139]. The effect of naringin has also been evaluated in orchidectomized male rats and it increased the levels of antioxidant status, plasma IGF-1, bone mineral density and calcium contents in the femur and lumbar; at the same time, the fecal and urinary excretion of calcium, as well as the plasma TRAP activity, were suppressed [140]. In senescent male rats, dietary supplementation with naringin resulted in an improved bone mineral density at the distal metaphyseal area as well as lowered deoxypyridinoline level, but bone formation did not appear to be affected (no effect on osteocalcin was observed, with modest modulation of tibial BMP-2 mRNA expression) [141]. Using a titanium-induced diabetic mouse model of calvarial osteolysis, treatment with naringin promoted new bone formation as demonstrated by elevated calvaria thickness, bone volume, midline suture area, and osteocalcin level [142]. In a study of the osteogenesis potential of the human periodontal ligament stem cells, a transplant of the naringin-treated cells in mice showed the presence of early osteoblast development and trabecular bone tissue, in which expressions of bone γ-carboxyglutamate protein (BGP) and osteopontin were detected [132].

The potential use of naringin in bone healing and repair has also been explored. Grafts containing naringin and collagen matrix for the treatment of experimental bone defects in rabbits suggested that the compound could promote new bone formation [143]. An implant of porous gelatin composite containing naringin was found to enhance osteogenic proliferation and nodule formation in the rabbit calvarial bone [144]. In a controlled-release nanoscaffold model incorporated with naringin, it supported osteoblast adhesion, proliferation, differentiation, and mineralization of MC3T3-E1 cells; and the same nanoscaffold suppressed osteoclast formation in a mouse calvarial critical size defect organ culture model [145]. In a study to determine the effect of naringin on wear debris-associated osteolysis in RAW 264.7 cells, naringin treated with polymethylmethacrylate particles could suppress osteoclastogenesis induced by the particles [146]. When the polymethylmethacrylate particles were implanted on the calvariae of mice followed by treatment with naringin, results demonstrated a suppression of osteolysis [135]. Using the mouse air pouch model, local injection of naringin ameliorated the particle-induced inflammatory tissue response and subsequent bone resorption [146,147]. Oral treatment with naringin in a tibia pin-implantation mouse model also demonstrated suppression of periprosthetic bone resorption [147].

Apart from the above flavonoids, phenolic compounds [including phloroglucinol, protocatechuic acid ethyl ester, 2-amino-3,4-dimethyl benzoic acid, 3-(3,5-dimethyl-pyrazol-1-yl)-benzoic acid, chlorogenic acid, syringic acid, trans-ferulic acid, (−)-epigallocatechin, epigallocatechin gallate, quercetin dehydrate, luteolin and emodin] have been identified from a fraction of Drynariae Rhizoma that exhibited anti-osteoporotic activity [148].

### 3.7. Ecliptae Herba (Above-Ground Parts of Eclipta prostrata L.)

Ecliptae Herba, “Mo-Han-Lian” in Chinese medicine, has tonifying effects on liver and kidney and is used as a tonic agent to treat problems arising during aging, such as fatigue and knee weakness. It is often prescribed, in combination with other herbs, for the treatment of menopausal syndrome [149].

**Figure 6:**
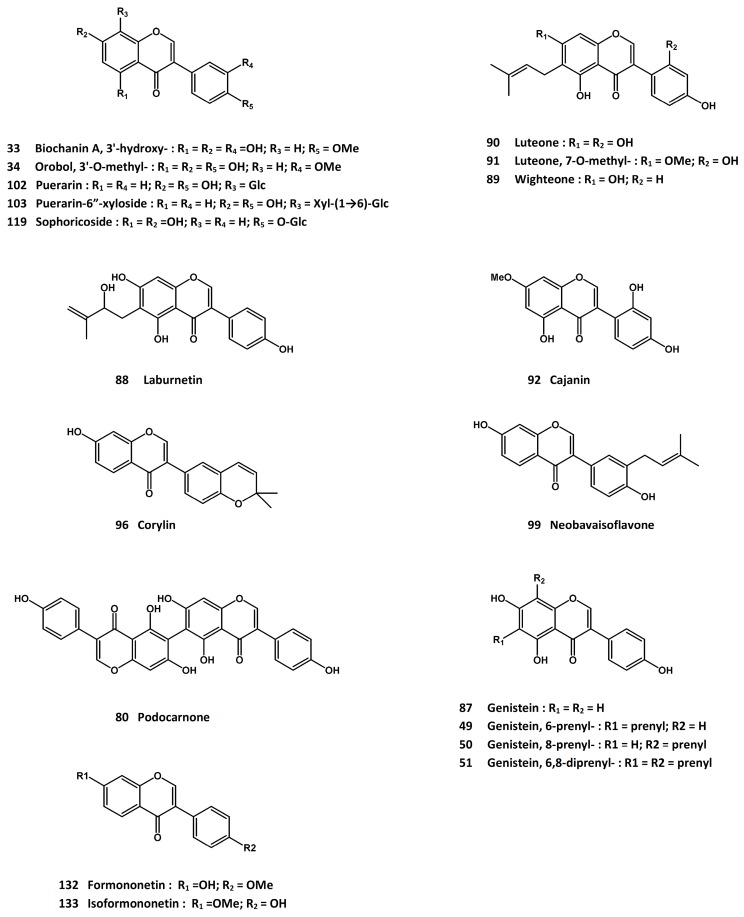
Isoflavonoids Reported to Possess Osteoprotective Property.

A bioactivity-guided isolation has led to the identification of diosmetin (**32**, Figure 1), 3’-hydroxybiochanin A (**33**, Figure 6), and 3’-*O*-methylorobol (**34**, Figure 6) as active principles to increase the alkaline phosphatase activity in primary culture of mouse osteoblasts [150]. The volatile fraction and ethanolic extract of the herbal drug displayed stimulatory activity on osteoblast proliferation and alkaline phosphatase activity [151]. In ovariectomized rats, treatment with an aqueous extract of *E. prostrata* led to down-regulation of RANKL expression and decreased serum interleukin-6 levels, together with an elevation of serum calcitonin. No effects on osteoprotegerin and parathyroid hormone were observed in the study [152].

Echinocystic acid (**35**, Figure 3) is an anti-inflammatory [153,154] and anti-hepatitis C virus ingredient [155] of *E. prostrata*. The osteoprotective property of the compound was recently shown in ovariectomized rats, in which echinocystic acid improved trabecular architecture, as evidenced by higher levels of bone volume/tissue volume ratio, trabecular number and trabecular thickness, as well as lower levels of trabecular separation and structure model index. The biochemical marker profile was also improved by elevations of osteocalcin, alkaline phosphatase, deoxypyridinoline, and urinary calcium and phosphorus levels. At the same time, the serum levels of IL-1β and TNF-α decreased [156].

Another compound, wedelolactone (**36**, Figure 4), was obtained from the ethyl acetate extract of *E. prostrata* and shown to inhibit osteoclast proliferation and differentiation. Thus, the compound inhibited RANKL-induced TRAP activity and reduced the number of multinucleated osteoclast-like cells when tested in the RAW 264.7 cell line [157].

### 3.8. Epimedii Folium [Leaf of Epimedium brevicornum Maxim., E. sagittatum (Siebold and Zucc.) Maxim., E. pubescens Maxim. or E. koreanum Nakai]

Epimedii Folium, “Yin-Yang-Huo” in Chinese medicine, is a liver- and kidney-tonic often used for the treatment of general physical weakness, discomfort and weakness in joints (e.g., back and knees) and arthritic pain. The *Epimedium* herbs are also allegedly aphrodisiacs. A number of medicinal formulas for treating bone disorders contain Epimedii Folium as major ingredient, and some of them have demonstrated *in vitro* and *in vivo* activities in experimental studies [158].

A variety of chemical ingredients have been found in Epimedii Folium, including flavonoids, lignans, ionones and terpenoids; by far the number of flavonoids exceeds other chemical types. Interestingly, many of the *Epimedium* flavonoids are phytoestrogens and they contain a prenyl (pentenyl) group at the C-8 position; some are glycosylated at other positions as well [159]. In ovariectomized rats, the flavonoid fraction of the plant drug has shown inhibitory activity against bone resorption and stimulating activity in bone formation [160]. The total flavonoid fraction also exerted anabolic effect in ovariectomized rats by promoting osteogenic activity (as indicated by increased serum levels of osteocalcin and bone mineral density, bone volume/tissue volume ratio, trabecular number, as well as improved bone histomorphometric parameters) and suppressing adipogenic differentiation of bone marrow stromal cells [161]. The total flavonoids of *Epimedium* have also been found to decrease mRNA expression levels of fat generation factors such as peroxisome proliferator activated receptor gamma 2 (PPARγ-2) and CCAAT enhancer-binding protein-α (C/EBPα), while promoting osteoblast differentiation in bone marrow stromal cells obtained from ovariectomized rats [162]. In a 24-month randomized double-blinded placebo-controlled clinical trial in postmenopausal women, *Epimedium*-derived flavonoids improved bone mineral density in the femoral neck and lumbar spine together with a decrease in urinary levels of deoxypytidinoline [163].

Among the chemical constituents of *Epimedium*, icariin (**37**, Figure 1), a prenylated flavonol diglycoside, is most studied for its potential applications in promoting bone health and other pharmacological effects [158,164,165]. In ovariectomized rats, icariin displayed anti-osteoporotic activity as demonstrated by increased bone density [166], improved bone biomechanical strength and histopathological parameters [167,168], as well as an increase in the mRNA expression ratio of osteoprogeterin (OPG)/RANKL in tibia [169]. The involvement of bone mesenchymal stem cell differentiation and increase in the secretion of early osteoblast differentiation factors such as osteocalcin, collagen 1, and Runt-related transcription factor 2 (Runx2) were demonstrated in animal models [170]. While icariin could protect ovariectomized animals from bone loss, it lowered adipogenesis in bone marrow as well [171]. Icariin also protected against *in vivo* glucocorticoid (dexamethasone)-induced osteoporosis and *in vitro* glucocorticoid-induced osteocyte apoptosis [172]. After feeding dexamethasone-treated mice with icariin, serum calcium was increased together with a decrease in urine calcium. Icariin reversed trabecular deteriorations and stimulated bone remodeling, as evidenced by increased osteoprogeterin (OPG) and FGF-23 and decreased CTX and TRAP-5b levels. In the same experiment, suppression of the mRNA expressions of MMP-9 and CAII in tibia was also observed [173]. In a study to demonstrate the role of osteoprogeterin on the activity of icariin, the anabolic and anti-resorptive effects of icariin on trabecular bone were found to diminish in an OPG-knockout mouse model [174]. In contrary, however, a more recent study using OPG-knockout mice demonstrated that icariin was able to stimulate new bone formation and prevent OPG-deficient-induced bone loss [175]. Activation of the target genes of β-catenin signaling (such as AXIN2, DKK1, TCF1, and LEF1) was observed, leading to the proposal that the Wnt/β-catenin-BMP signaling was involved [175]. In a study to examine the effects of icariin on signal messengers, the osteogenic activity was related to the PI3K-AKT-eNOS-NO-cGMP-PKG signaling pathway [176]. A recent study demonstrated that, when the bone marrow stromal cells obtained from ovariectomized rats were transplanted into nude mice, treatment with icariin could restore the osteogenic differentiation and mineralization of the ovariectomy-derived stem cells [177].

*In vitro* studies also support the osteoprotective potential of Epimedii Folium. Flavonoid ingredients of *Epimedium* such as icariin, epimedin B (**38**, Figure 1) and epimedin C (**39**, Figure 1) were found to possess proliferative property in osteoblast cell lines such as the osteoblast-like UMR-106 cells [178], rat bone mesenchymal stem cells, and human bone mesenchymal stem cells [179,180]. The *in vitro* stimulatory effect on osteogenic proliferation and differentiation was shown to be associated with the up-regulation of BMP-2 [180,181,182,183,184,185], BMP-4 [186], Runx2 [183,186,187,188], collagen 1α2 [183,188], osterix (Osx) [183,187,188] inhibitor of DNA-binding 1 (Id-1) [186], Smad4 [181,185], Cbfa1/Runx2 [181,185], OPG [181,185], and nitrogen oxide production [181], as well as down-regulation of RANKL [181,187]. Icariin did not activate estrogen response element (ERE)-luciferase activity in UMR-106 cells, but it increased estrogen receptor α phosphorylation at Ser118 [169,189]. The anabolic effect was attributed to the activation of estrogen receptor involving ERK and JNK pathway [190]. In a study using interleukin-1β-stimulated SW1353 chondrosarcoma cells, icariin decreased the levels of receptor activator of nuclear factor-κB (RANK) and RANKL, together with an up-regulation of phosphorylated-Erk1/2 and down-regulation of phosphorylated p38. Its regulation on OPG-RANKL-RANK was therefore proposed to be mediated through the mitogen-activated protein kinase (MAPK) pathway [191,192]. Using a rat model of corticosterone-induced osteoporosis, feeding with icariin resulted in an alteration of mRNA expressions of 11 genes (compared to the normal levels) in the bone marrow stromal cells; five of these genes were involved in osteoblast differentiation, cell cycle regulation and the Notch signal pathway [193]. Under hypoxic conditions, icariin was able to attenuate oxidative stress and apoptosis in rat calvarial osteoblasts, while preserving the osteogenic potential (as revealed by increased levels of Runx2, oxterix and BMP-2 gene expression and alkaline phosphatase activity) [194]. In a search for potential treatment of steroid-associated osteonecrosis of femoral head, icariin was found to induce P-glycoprotein expression, decreased oxidative stress, and promoted osteogenesis in bone marrow stem cells obtained from patients with steroid-associated osteonecrosis of femoral head [195].

*In vitro* studies also support the osteoprotective potential of Epimedii Folium. Flavonoid ingredients of *Epimedium* such as icariin, epimedin B (**38**, Figure 1) and epimedin C (**39**, Figure 1) were found to possess proliferative property in osteoblast cell lines such as the osteoblast-like UMR-106 cells [178], rat bone mesenchymal stem cells, and human bone mesenchymal stem cells [179,180]. The *in vitro* stimulatory effect on osteogenic proliferation and differentiation was shown to be associated with the up-regulation of BMP-2 [180,181,182,183,184,185], BMP-4 [186], Runx2 [183,186,187,188], collagen 1α2 [183,188], osterix (Osx) [183,187,188] inhibitor of DNA-binding 1 (Id-1) [186], Smad4 [181,185], Cbfa1/Runx2 [181,185], OPG [181,185], and nitrogen oxide production [181], as well as down-regulation of RANKL [181,187]. Icariin did not activate estrogen response element (ERE)-luciferase activity in UMR-106 cells, but it increased estrogen receptor α phosphorylation at Ser118 [169,189]. The anabolic effect was attributed to the activation of estrogen receptor involving ERK and JNK pathway [190]. In a study using interleukin-1β-stimulated SW1353 chondrosarcoma cells, icariin decreased the levels of receptor activator of nuclear factor-κB (RANK) and RANKL, together with an up-regulation of phosphorylated-Erk1/2 and down-regulation of phosphorylated p38. Its regulation on OPG-RANKL-RANK was therefore proposed to be mediated through the mitogen-activated protein kinase (MAPK) pathway [191,192]. Using a rat model of corticosterone-induced osteoporosis, feeding with icariin resulted in an alteration of mRNA expressions of 11 genes (compared to the normal levels) in the bone marrow stromal cells; five of these genes were involved in osteoblast differentiation, cell cycle regulation and the Notch signal pathway [193]. Under hypoxic conditions, icariin was able to attenuate oxidative stress and apoptosis in rat calvarial osteoblasts, while preserving the osteogenic potential (as revealed by increased levels of Runx2, oxterix and BMP-2 gene expression and alkaline phosphatase activity) [194]. In a search for potential treatment of steroid-associated osteonecrosis of femoral head, icariin was found to induce P-glycoprotein expression, decreased oxidative stress, and promoted osteogenesis in bone marrow stem cells obtained from patients with steroid-associated osteonecrosis of femoral head [195].

The osteogenic activity of icariin has made it a promising agent for bone tissue engineering [196]. Thus, in a mouse calvarial defect model, transplants containing a mixture of icariin and calcium phosphate cement led to significant new bone and blood vessel formation, suggesting potential use for bone inductive tissue engineering [182,196]. The icariin-loaded porous β-tricalcium phosphate ceramic disks were shown to be favorable to supporting the proliferation and differentiation of Ros17/28 cells [197]. An icariin/tricalcium phosphate porous scaffold enhanced new bone and vascular formation after 12 weeks of treatment in a rabbit model of femoral head osteonecrosis [198]. Delivery porous PHBV scaffolds containing icariin were found to enhance the proliferation of human osteoblast-like MG-63 and the pre-osteoblast MC3T3-E1 cells [199]. Icariin displayed *in vitro* inhibitory activity on inflammatory osteoclastogenesis in RAW264.7 cells induced by titanium particles, suggesting that it might be useful for the prevention and treatment of wear particle-induced osteolysis occurred after joint replacement [200]. Such protective effect was further demonstrated in a mouse calvarial model of titanium particle-induced osteolysis [201]. In addition, its potential application in cartilage tissue engineering was suggested by the observation that icariin promoted chondrogenic differentiation of bone marrow stem cells but had no effect on hypertrophic differentiation [202]. In another study, icariin was added into cell-hydrogel constructs derived from neonatal rabbit chondrocytes and collagen type 1, and such an icariin-containing construct accelerated the formation of chondroid tissue, improved the restoration efficiency of supercritical-sized osteochondral defects in rabbit, and enhanced the integration of new cartilage with subchondral bone [203]. When a drug delivery system composed of icariin, vancomycin and injectable calcium phosphate cement was implanted in rabbits suffering from Staphylococcus- contaminated bone defects, the bone defects were completely repaired after 12 weeks, showing the potential for treating contaminated bone injury or infectious bone diseases [204]. In a study of the effect of combined mechanical strain and icariin treatment on osteogenic proliferation and differentiation in MC3T3-E1 cells, the combination was found to be able to activate the nuclear factor κ-light-chain-enhancer of activated B cells (NF-κB) pathway to improve the cellular proliferation and differentiation better than icariin treatment alone [205].

Icariin inhibited bone resorption both *in vitro* and *in vivo* [206,207]. The anti-osteoclastic property was demonstrated by inhibition of osteoclast formation induced by RANKL and macrophage colony-stimulating factor (M-CSF) in mouse bone marrow culture [208]; it also suppressed osteoclast differentiation in both osteoblast-preosteoclast co-culture and osteoclast progenitor cell culture, and reduced motility and bone resorption activity in isolated osteoclasts [209]. Icariin suppressed the osteoclast differentiation marker TRAP, IL-6, tumor necrosis factor (TNF)-α, RANKL, as well as the synthesis of cyclooxygenase-2 and prostaglandin E2. The anti- osteoclastic activity seemed to be related to the suppression of the p38 and c-Jun N terminal kinase (JNK) pathway [210].

Besides icariin, a number of flavonol constituents of *Epimedium* are also osteo-active. Thus, epimedins A (**40**, Figure 1), B (**38**, Figure 1), C (**39**, Figure 1) and icariin were demonstrated to interact with MC3T3-E1 cells in a biomembrane extraction model [211], and epimedin B and epimedin C promoted the proliferation of UMR-106 cells [178]. In a zebrafish model of anti-osteoporosis, epimedin A and baohuoside-1 (icarisinde II) (**41**, Figure 1) were found to be active [212]. Baohuoside-1 also suppressed the formation and activity of osteoclasts by inhibiting proliferation and differentiation, inducing apoptosis and cell cycle arrest, and suppressing bone resorption [213]. In osteoblast-like UMR-106 cells, baohuoside-1 stimulated cell proliferation rate, alkaline phosphatase activity, and OPG/RANKL mRNA expressions [189]. Maohuoside A (**42**, Figure 1) isolated from E. koreanum was found to promote osteogenesis of rat and mouse mesenchymal stem cells via the BMP and MAPK signaling pathways [214,215]. Sagittatoside A (**43**, Figure 1) selectively activated ERE-luciferase activity via estrogen receptor α and it induced ER-α phosphorylation at serine-118 residue [189]. Both compounds seemed to exert their actions by ligand-independent activation of ER-α. Ikarisoside A (**44**, Figure 1) was shown to inhibit osteoclastogenic differentiation in RAW 264.7 cells via JNK and NF-κB signaling pathways [216].

Icariin is metabolized into a number of metabolites, including icaritin (**45**, Figure 1), icariside I (**46**, Figure 1), icariside II (baohuoside-1) (**41**, Figure 1), desmethylicaritin (**47**, Figure 1), as well as anhydroicaritin (**48**, Figure 1) and its glycosides [217,218]. Both icariside II and icaritin enhanced the differentiation and proliferation of osteoblasts and facilitated matrix calcification; they also inhibited osteoclastic differentiation and reduced the motility and bone resorption activity of osteoclasts [209]. Icariside II was reported to display higher potency than icariin in promoting osteoblast proliferation and differentiation [219]. The osteogenic activity of icariside II could be blocked by estrogen receptor inhibitor ICI-182780, indicating the involvement of estrogen signaling pathway, although icariin itself may act through non-estrogenic mechanisms [219]. On the other hand, icaritin was able to enhance osteoblastic differentiation of mesenchymal stem cells (as indicated by increased mRNA expressions of relevant markers of osteoblastogenesis) [220,221]; it also inhibited adipogenesis, which was associated with the suppression of glycogen synthase kinase-3β (GSK3β) and peroxisome proliferator-activated receptor γ (PPARγ) [221]. Icaritin was shown to possess anabolic and anti-resorptive properties on osteoporotic bone in ovariectomized rats, but the beneficial effects seemed to be dependent on the intervention timing relative to estrogen depletion. Rats treated with icaritin one month after ovariectomy were protected, but those treated at 3-month post-operation did not respond to the effects of icaritin [222]. The compound was also found to reduce the incidence of steroid-associated osteonecrosis with inhibition of intravascular thrombosis and extravascular lipid-deposition [223]. Icaritin was shown to display synergistic effect with icariside II in suppressing the growth of pre-osteoclastic RAW 264.7 cells [224].

Icaritin has been studied for potential applications in fabricated scaffold materials for bone healing by incorporating the compound into a porous poly(l-lactide-co-glycolide)/tricalcium phosphate (PLGA/TCP) scaffold [225]. The structure, composition, and mechanical properties of the scaffold were characterized and it was found to facilitate the attachment, proliferation and osteogenic differentiation of bone marrow mesenchymal stem cells [226,227]. When tested *in vivo* in rabbit bone tunnel model, muscle pouch model, or an ulnar bone defect model, the scaffold promoted new bone formation within the bone defect and enhanced new vascularization in the rabbit muscle pouch experiment [228,229]. The potential application of such scaffold for the prevention of hip joint collapse was also explored [230].

### 3.9. Erythrina variegata L.

The bark of *Erythrina variegata* is a folkloric medicine used for treating arthritic pain.

Administration of an extract to ovariectomized rats for 14 weeks resulted in an increase in serum osteoprotegrin, alkaline phosphatase and urinary deoxypyridinoline levels [231]. Histomorphometric analysis of the proximal end of the tibia showed the prevention of estrogen deficiency-induced decrease in trabecular thickness and trabecular area, as well as restoring the increase in trabecular separation in a dose-dependent manner [231]. It also suppressed the up-regulation of cathepsin K mRNA and the down-regulation of osteoprotegrin mRNA in the tibia [232]. In vitro studies indicated that the plant extract decreased TRAP-positive cell numbers in RANKL-treated RAW 264.7 cells [232]. The protective effects on bone properties were likely mediated by inhibiting bone resorption via the suppression of osteoclast differentiation and maturation. A series of prenylated isoflavones such as 6-prenylgenistein (**49**, Figure 6), 8-prenylgenistein (**50**, Figure 6), and 6,8-diprenylgenistein (**51**, Figure 6) were isolated from the active fractions, and a structure-activity relationship analysis indicated the prenylation at C-8 was most active in promoting UMR-106 cell proliferation, differentiation and mineralization [233].

### 3.10. Eucommiae Cortex (Stem bark of Eucommia ulmoides Oliv.)

Eucommiae Cortex is derived from the stem bark of *Eucommia ulmoides*. It is a common liver- and kidney-tonic often used in Chinese medicine for strengthening general physical weakness, muscle pain, and bone disorders such as low back pain and weakness in the knees and other joints.

In ovariectomized rats fed with Eucommiae Cortex extract, the femur biomechanical quality and trabecular microarchitecture were improved without hyperplastic effect on uterus [234]. When adolescent female rats were treated with the bark extract, promotion of longitudinal bone growth was observed, with increased BMP-2 and IGF-1 expressions in the proliferative and hypertrophic zones [235]. In a disuse-induced osteoporosis model of hind-limb suspended rats, the *Eucommia* bark extract could prevent bone loss as indicated by decreased levels of bone turnover markers; it enhanced the biomechanical bone strength and prevented the deterioration of trabecular bone microarchitecture [236].

**Figure 7:**
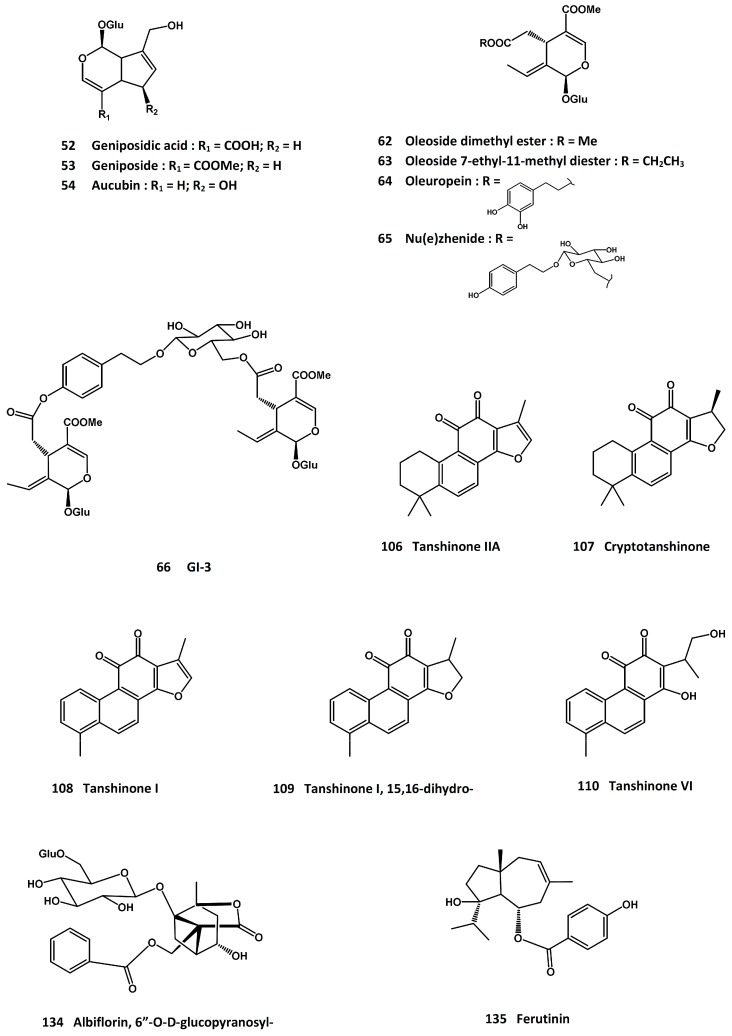
Monoterpenoids, Sesquiterpenoids and Diterpenoids Reported to Possess Osteoprotective Property.

Three of the iridoid glycoside ingredients, geniposidic acid (**52**, Figure 7), geniposide (**53**, Figure 7) and aucubin (**54**, Figure 7), exhibited proliferative activity in osteoblasts; and at the same time, they suppressed the growth of osteoclasts [237]. The *in vitro* and *in vivo* osteoprotective effects have also been ascribed to the lignan constituents [238]. In addition, 5-hydroxymethyl-2-furaldehyde (**55**, Figure 4) was reported to enhance the osteogenic differentiation of rat bone mesenchymal stem cells [239].

In an attempt to explore the potential utilization of other plant parts of *E. ulmoides*, an ethanol extract of the leaves were found to promote the growth of MC3T3-E1 cells and suppress the H2O2-indcued apoptosis [240]. It also prevented ovariectomy-induced osteoporosis and obesity in rats [241]. On the other hand, the total glycosides obtained from the seeds of *E. ulmoides* increased bone mineral density, bone volume/tissue volume ratio, connectivity density, trabecular number, and trabecular thickness in normal rats [242].

### 3.11. Ligustri Lucidi Frustus (Fruit of Ligustrum lucidum W.T. Aiton)

Ligustri Lucidi Fructus is a tonic often included in Chinese herbal prescriptions for vitalizing the “liver and kidney” functions; it is indicated for “weakness of the loin and knees”, which may represent a symptom of bone deterioration.

A recent review article on the anti-osteoporosis activity of *Ligustrum lucidum* fruit has summarized the current knowledge about this herb [243]. Several ingredients have been demonstrated to display potential anti-osteoporosis activities in cell-based and/or animal models. Thus, oleanolic acid (**56**, Figure 3) and its glycosidic and synthetic derivatives have been known to be inhibitors of osteoclast formation [244,245,246,247]. They inhibited the formation of osteoclast-like multinucleated cells induced by 1α, 25-dihydroxyvitamin D3. Apart from the anti-osteoclastogenic activity, oleanolic acid was able to promote osteoblastic differentiation and change the gene expression profile of bone marrow stromal cells obtained from corticosterone-induced osteoporotic rats [193]. In ovariectomized rats, oleanolic acid exerted osteoprotective effect by increasing the population of osteoblasts as well as the levels of osteocalcin and the Runt-related protein-2. It also stimulated the osteoblastic differentiation of bone mesenchymal stem cells *in vitro*. Gene expression profile analysis suggested that the effect might be related to the Notch signaling pathway [248].

Oleanolic acid acetate was demonstrated to inhibit receptor activator of nuclear factor-κB (RANKL)-induced osteoclast differentiation; and it attenuated lipopolysaccharide-induced bone erosion in mice [249].

Another triterpene ingredient of *L. lucidum*, ursolic acid (**57**, Figure 3), has been found to stimulate osteoblast differentiation and mineralization by activating osteoblast-specific genes such as mitogen-activated protein kinases, nuclear factor-κB, and activator protein-1 [250]. It also promoted bone formation in a mouse calvarial bone formation model [251]. In addition, recent studies showed that ursolic acid was able to inhibit RANKL-induced osteoclast differentiation and down-regulate the NFATc1-regulated osteoclast marker genes [252,253]. Using a mouse model of titanium particle-induced osteolysis, ursolic acid protected calvarial bone loss and decreased the population of tartrate-resistant acid phosphatase (TRAP)-positive osteoclasts [253].

Two screening and bioactivity-guided isolation reports on the osteoprotective activity of *L. lucidum* have identified a number of active compounds [254,255]. While the screening results are considered to be preliminary at this time, the active compounds were reported to promote the proliferation of osteoblast-like UMR-106 cells, increase the alkaline phosphatase activity, and/or protect the cells from hydrogen peroxide-induced damage. The active compounds include tyrosol (**58**, Figure 4), hydroxytyrosol (**59**, Figure 4), salidroside (**60**, Figure 4), acteoside (**61**, Figure 2), oleoside dimethyl ester (**62**, Figure 7), oleoside-7-ethyl-11-methyl diester (**63**, Figure 7), oleuropein (**64**, Figure 7), nu(e)zhenide (**65**, Figure 7), GI-3 (**66**, Figure 7), luteolin 7-*O*-β-d-glucopyranoside (**67**, Figure 7), apigenin (**68**, Figure 1), apigenin 7-*O*-β-d-glucopyranoside (**69**, Figure 1), and apigenin 7-*O*-β-d-(6”-*O*-acetyl)-glucopyranoside (**70**, Figure 1). Among these compounds, tyrosol, hydroxytyrosol and oleuropein are also present in the olive oil, which has been reported to possess anti-osteoporosis property [256]; and salidroside was active in suppressing diabetes-related osteoporosis in animal model [257].

### 3.12. Morindae Officinalis Radix (Root of Morinda officinalis F.C. How)

Morindae Officinalis Radix (“Ba-Ji-Tian” in Chinese medicine) is a well-known tonic for the Yang component of the kidney. It is renowned for use to strengthen tendon and bone, as well as to alleviate arthritis. It is also used to treat menstrual disorder and female infertility.

Using a neurectomized disused osteoporotic mouse model, feeding with *Morinda* extract resulted in both suppression of bone resorption and enhancement of bone formation. Thus, the thickness of the hind-limbs, tibia failure load, tibia bone mineral density, tibia calcium and phosphorus contents, and serum osteocalcin levels were elevated. In addition, the histomorphometrical parameters of the tibia such as volume, length and thickness of trabecular bone and thickness of cortical bone were improved after treatment [258]. The polysaccharide-rich fraction of the plant was shown to increase bone mineral density and decrease serum IL-6 and TNFα levels in ovariectomized rats [259]. However, another study reported that, an ethanol extract of *Mordina officinalis* roots increased trabecular bone mineral content and bone mineral density, the phosphorus and calcium levels and OPG, as well as suppressed the levels of TRAP, ACTH and corticosterone, it did not reverse the levels of alkaline phosphatase, IL-6, and TNFα [260]. The controversy has yet to be solved. The active ingredients that inhibited osteoclastic bone resorption include physicion (**71**, Figure 4), rubiadin (**72**, Figure 4), rubiadin-1-methyl ether (**73**, Figure 4), 2-hydroxy-1-methoxy-anthraquinone (**74**, Figure 4), 1,2-dihydroxy-3-methylanthraquinone (**75**, Figure 4), 1,3,8-trihydroxy-2-methoxyanthraquinone (**76**, Figure 4), 2-methoxy-3-hydroxyanthraquinone (**77**, Figure 4), 2-methoxyanthraquinone (**78**, Figure 4), and scopoletin (**79**, Figure 4) [261,262].

### 3.13. Podocarpium podocarpum (DC.) Yang et Huang [syn. Desmodium podocarpum DC.]

The whole plant of *Podocarpium podocarpum* is occasionally used in Chinese folk medicine for the treatment of fever and cough.

The plant is known to contain cytotoxic phenylpropanoids [263] and flavonoids [264,265]. An ethanol extract of the plant exhibited anti-osteoporosis activity in ovariectomized rats, as shown by inhibition of urinary calcium excretion and the activities of bone resorption markers such as TRAP, cathepsin K, and deoxypyridinoline crosslinks; the bone quality (e.g., bone mineral content, bone volume fraction, connectivity density, tissue mineral content, tissue mineral density, and trabecular number) was improved [265]. When the isolated flavonoids were evaluated in osteoblasts and osteoclasts, several of them exhibited stimulatory activity to enhance osteoblast proliferation, increase alkaline phosphatase activity, and promote mineralized nodes formation; at the same time, they suppressed osteoclastic TRAP activity. The active compounds include podocarnone (**80**, Figure 6), luteolin (**81**, Figure 1), astragalin (**82**, Figure 1), afzelin (**83**, Figure 1), kaempferitrin (**84**, Figure 1), rutin (**85**, Figure 1), quercetin-7-*O*-d-glucopyranoside (**86**, Figure 1), genistein (**87**, Figure 6), laburnetin (**88**, Figure 6), wighteone (**89**, Figure 6), luteone (**90**, Figure 6), and 7-*O*-methyl-luteone (**91**, Figure 6) [265]. Cajanin (**92**, Figure 6) was also reported to promote osteoblast differentiation involving the MEK-ERK and Akt pathways, and it increased bone mineral density, bone biomechanical strength, mineral apposition rate and bone formation rate in newborn female rats [266].

### 3.14. Psoraleae Fructus (Fruit of Psoralea corylifolia L. [syn. Cullen corylifolium (L.) Medik.])

Psoraleae Fructus, literally meaning “bone-marrow tonic” in Chinese medicine, is a well-known herbal drug for kidney-tonifying and bone-nourishing applications. The herbal drug is often used in the treatment of general physical weakness, joint disorders, lower back pain and knee weakness.

Ovariectomized rats fed with *Psoralea* extract for three months showed an increase in serum calcium and a concomitant decrease in urinary calcium excretion, suppression of the upregulated serum osteocalcin level, and increase in bone mineral density [267]. In male mice, feeding with *Psoralea* extract led to an increase in bone volume/tissue volume ratio; the bone trabeculae also increased in thickness so that bone density was increased [268].

*P. corylifolia* is well known to contain furocoumarins such as psoralen (**93**, Figure 4) and isopsoralen (**94**, Figure 4) [269,270]. Psoralen is an estrogen receptor-α agonist [271] and a photoactive mutagen [270]. It has been used in photochemotherapy together with long-wavelength ultraviolet irradiation (PUVA) for treating psoriasis, vitiligo and other skin problems. As far as bone disorders are concerned, psoralen, when mixed with collagen matrix, was shown to stimulate local new bone formation in the defected areas of a rabbit model of bone grafting, the outcome being better than those grafted with collagen matrix alone [272]. Similar result was observed with the plant extract [273]. In ovariectomized rats, psoralen improved bone mass indicators including increased trabecular thickness and decreased trabecular space. The osteoprotective effect was pointed to an association with the Notch signaling pathway, thus possibly related to the stimulation of differentiation of bone mesenchymal stem cells [274]. Using a rat model of corticosterone-induced osteoporosis, feeding with psoralen resulted in an alteration of mRNA expressions of 12 genes (compared to the normal levels) in the bone marrow stromal cells; five of these genes were involved in osteoblast differentiation, cell cycle regulation and the Notch signal pathway [193]. *In vitro* studies have demonstrated that psoralen promoted osteoblast differentiation in primary mouse calvarial osteoblasts as evidenced by up-regulation of expressions of type-1 collagen, osteocalcin and bone sialoprotein [275]. The action was likely associated with the activation of BMP signaling, including the expressions of BMP-2 and BMP-4 genes, phosphor-Smad1/5/8 protein, osterix, and BMP reporter (12xSBE-OC-Luc) activity [275]. The compound was also shown to work on cartilages; it promoted cartilaginous gene expressions (e.g., type-II collagen, aggrecan, and SOX-9) in rat chondrocytes [276] as well as in a tissue culture of rat cartilage of lumbar intervertebral disc [277].

Isopsoralen (**94**, Figure 4) has been reported to promote osteogenic differentiation of bone marrow stromal stem cells [278] and rat calvarial osteoblasts [279], as demonstrated by the elevation of alkaline phosphatase activity, calcium salt sediment yield, osteocalcin, and calcified tubercle amount.

Psoralidin (**95**, Figure 4) is a coumestan derivative present in *Psoralea corylifolia*. It was reported to be able to increase not only the bone density of lumbar vertebra and thigh bone of ovariectomized rats, but also the maximum bending strength [280].

Besides coumarins, two flavonoid components of *P. corylifolia*, corylin (**96**, Figure 6) and bavachin (**97**, Figure 5), were identified as active principles for osteoblastic stimulating activity in UMR-106 cells [281,282]. Bavachin and isobavachin (**98**, Figure 5) were demonstrated to stimulate rat calvarial osteoblast proliferation and differentiation as well [283].

Neobavaisoflavone (**99**, Figure 6) was found to promote osteogenesis in MC3T3-E1 cells as evidenced by enhancement of alkaline phosphatase activity, upregulation of bone-specific matrix protein expressions including type 1 collagen, osteocalcin and bone sialoprotein [284]. Neobavaisoflavone also up-regulated the expressions of bone-specific transcription factors such as Runx2 and osterix [284]. Activation of p38 phosphorylation was also observed. Taken together, the osteogenic activity of neobavaisoflavone might act through activation of p38-dependent signaling pathway to up-regulate the mRNA levels of Runx2 and osterix, thereby stimulating bone matrix proteins expression [284].

Bakuchiol (**100**, Figure 4) is a meroterpene found in *P. corylifolia*. It had strong binding affinity for estrogen receptor α. In ovariectomized rats, the compound reduced bone loss by increasing alkaline phosphatase, serum estradiol, and bone mineral density [285]. In mouse primary calvarial osteoblasts, bakuchiol enhanced cell differentiation [283].

Apart from the osteoblastic activities, *P. corylifolia* contains anti-osteoclastic constituents. Thus, bavachalcone (**101**, Figure 4) was found to inhibit osteoclast formation from precursor cells, suppressing the activation of MEK, ERK, and Akt (protein kinase B), as well as inducing c-Fos and NFATc1 [286].

### 3.15. Puerariae lobatae Radix [Root of Pueraria lobate (Willd.) Ohwi]

Puerariae lobatae Radix is the dried roots of *Pueraria lobata*. It is widely used in Chinese medicine as a “cooling” agent for the treatment of fever and other “hot” diseases. Although this herbal drug is not traditionally used for treating bone disorders, it has found applications for osteoporosis more recently due to the presence of isoflavonoids such as daidzein and genistein, both of which are well known soybean phytoestrogens. Since daidzein and genistein are beyond the scope of this review, only other *Pueraria* isoflavonoids such as puerarin (**102**, Figure 6) will be discussed here. Puerarin is an isoflavone possessing a structure of daidzein-8-*C*-glucoside, and its general pharmacological effects have recently been reviewed [287,288].

Owing to the presence of phytoestrogen contents in Puerariae lobatae Radix, the plant has been found to be able to prevent bone loss [289,290], enhanced bone mass, and promote osteoblast proliferation and differentiation [291,292,293] in ovariectomized animals. In a parietal bone defect model of rabbit, treatment with a graft containing puerarin and collagen matrix stimulated new bone formation [294]. Stimulation of new bone formation was also observed in a rat model of osteoblast implant [295]. In the streptozotocin-induced diabetic rat model, puerarin treatment suppressed the caspase-3 expression in osteoblasts and improved bone mineral density [296]. Puerarin prevented osteonecrosis induced by alcohol in mice as well as in cultured bone marrow stromal cells [297]. While the *in vitro* activity of puerarin has been shown to be dependent on estrogen receptors (see below), a report on feeding puerarin diet to ovariectomized mice suggested the anti-osteoporotic action was non-estrogen receptor mediated [298]. The osteoclast-inhibitory activity of puerarin was also reported [299].

*In vitro* osteogenic properties of puerarin were also documented; it increased cell viability, alkaline phosphatase activity, and mineral nodules formation in newborn rat osteoblasts [291,295,300,301]. The osteogenic activity has been proposed to be associated with the PI3K/Akt [300,302], p38 MAPK, and Wnt/β-catenin pathways, and the activity could be blocked by estrogen receptor antagonist ICI 182780 [291,302]. Puerarin was also shown to exert anti-apoptotic activity on osteoblast via the estrogen receptor-dependent ERK signaling pathway [303]. Using primary osteoblasts obtained from female mice, bone anabolic activity of puerarin was demonstrated and the osteogenic effect was induced by BMP-2 and NO synthesis, subsequently regulating Cbfa1/Runx2, osteoprotegerin, and RANKL gene expressions [304]. The involvement of interleukin-6 mediated by estrogen receptor α [305], and the NO/cGMP [306] pathways was also suggested. In MC3T3-E1 cells, puerarin promoted cell proliferation which might be mediated by activation of the TGF-β1/Smad pathway [307]. In a study using rat osteoblast-like UMR-106 cells, osteoblast differentiation, but not cell proliferation, was observed, and the activity was estrogen receptor dependent [308]. In an attempt to study the osteogenic activity of puerarin in non-human primate cells, baboon osteoblasts were found to be responsive to puerarin by displaying increased rate of proliferation and elevated mRNA levels of alkaline phosphatase and type-1 collagen, together with a decrease in the RANKL/OPG ratio [309]. Puerarin was recently shown to reduce the alveolar bone loss and collagen destruction in a rat model of ligature-induced periodontitis by inhibiting the production of RANKL, IL-1β, TNF-α, MMP-2 andMMP-9 [310].

Puerarin 6”-xyloside (**103**, Figure 6) has also been shown to possess anti-osteoporotic property on ovariectomized mice [311].

### 3.16. Rehmanniae Radix [Root of Rehmannia glutinosa (Gaertn.) DC.]

Rehmanniae Radix is the dried roots of *Rehmannia glutinosa*; it is available in Chinese medicine either raw (dried) or pre-treated with a steaming process. The steamed root is often used as a tonic for its liver- and kidney-tonifying effects, and it has a long history of medicinal applications for the treatment of joint weakness and arthritic pain.

An early *in vitro* study using osteoblasts demonstrated that the plant extract promoted the proliferation, the alkaline phosphatase activity, mRNA expressions of bone-related genes, and osteoprotegerin secretion [312] Its effects on osteoclasts, on the other hand, included decreased formation of the TRAP(+) multinucleated cells as well as decreased resorption areas in a culture of osteoclast precursors [312]. *In vivo* studies using ovariectomized rats revealed that the extracts alleviated the decreased trabecular bone mineral density and it increased the cortical bone thickness and the trabeculation of the bone marrow space [312]. The bone loss preventive effect of the plant was also demonstrated in ovariectomized rats after 8-week treatment [313]. The treated group showed significantly higher bone mineral density in the femur and lumbar when compared to the untreated ovariectomized animals.

Acteoside (verbascoside (**61**, Figure 2)), a caffeoyl phenylethanoid glucoside, was identified as an anti-resorption ingredient capable to reduce bone loss by blocking osteoclast activation [314]. The compound suppressed the effects of RANKL on osteoclast formation and differentiation from bone marrow macrophages and RAW264.7 macrophages, through the inhibition of transcription factors such as NF-κB, c-Fos and NFATc1. Furthermore, acteoside was found to increase the growth and differentiation of the UMR-106 [255] and MC3T3-E1 [315] cells; it also inhibited the X-ray irradiation-induced decrease in cell viability and DNA synthesis in MC3T3-E1 cells [316].

### 3.17. Salviae miltiorrhizae Radix et Rhizoma (Root and rhizome of Salvia miltiorrhiza Bunge)

The root and rhizome of *Salvia miltiorrhiza* has been used to treat gynecological disorders such as irregular menstruation, blood stasis, and abdominal pain.

In early studies, aqueous extracts of *S. miltiorrhiza* were reported to prevent trabecular bone loss in ovariectomized rats [317] and steroid-treated rats [318]. In a rat model of alloxan-induced diabetic osteoporosis, the plant extracts was shown to improve bone mineral density and increase the levels of alkaline phosphatase and TRAP [319]. Using a bone graph model in rabbits, the *S. miltiorrhiza* extract (mixed with collagen) increased bone formation by over 4-folds [320].

A number of chemical ingredients of *S. miltiorrhiza* have been shown to possess osteoprotective activities [321]. Salvianic acid A (**104**, Figure 2) increased bone formation markers including alkaline phosphatase and osteoprotegerin (OPG) in rat osteoblasts [322]. Salvianolic acid B (**105**, Figure 2) not only prevented bone loss in steroid-treated osteoporotic rats, but also increased bone mass and improve microvasculature in bones [323]. Tanshinone IIA (**106**, Figure 7) and cryptotanshinone (**107**, Figure 7) were reported to prevent trabecular bone loss in the lumbar vertebrae in ovariectomized rats, without evidence of demineralizing activity [324]. Indeed, tanshinone IIA and cryptotanshinone, as well as two other ingredients in S. miltiorrhiza, tanshinone I (**108**, Figure 7) and 15,16-dihydrotanshinone I (**109**, Figure 7), displayed *in vitro* activity against osteoclast differentiation [325,326], which might result from the suppression of genes such as calcitonin receptor, c-Src kinase, integrin β3, and c-Fos and NFATc1-induced RANKL, NF-κB, and COS-2/PGE2 [327,328,329]. More recently, another tanshinone derivative, tanshinone VI (**110**, Figure 7), was demonstrated to inhibit osteoclast differentiation by attenuating RANKL expression and NF-κB induction in a three-dimensional osteoblast/bone marrow model [330].

In a recent study of the cathepsin K inhibitory activity of *S. miltiorrhiza* extracts as a potential inhibitor of bone resorption, dihydrotanshinone and cryptotanshinone displayed anti-collagenase activity [331].

The above studies have revealed the inhibitory activity of *S. miltiorrhiza* on osteoclast differentiation without pinpointing the exact molecular mechanisms. In a study comparing the *in vitro* efficacy of the *Salvia* extract and four major ingredients (*i.e.*, tanshinone I, tanshinone IIA, cryptotanshinone, and 15,16-dihydrotanshinone I), the anti-osteoclastogenic activity of the extract was almost 1000 times higher than the sum of individual compounds. This suggested that the plant extract must have contained other unknown active ingredients, some of which might have synergistic effects with one another [326].

Apart from the anti-osteoclastogenic and anti-resorptive activities, the *S. miltiorrhiza* extract have demonstrated enhancing effect on bone remodeling by regulating the gene expression of alkaline phosphatase, OCN, OPG, and RANKL in MC3T3-E1 cells [332]. Salvianolic acids A and B have been reported to stimulate osteoblast differentiation and suppress adipogenic differentiation in prednisone-treated rats [322,323], likely acting through the activation of the ERK signaling pathway [333].

### 3.18. Sambuci Caulis (Stem of Sambucus williamsii Hance)

Sambuci Caulis, derived from the stems of *Sambucus williamsii*, is known in Chinese medicine as the “bone-healing wood”. It is often used for treating arthritic pain, joint disorders, traumatic damages and bone fracture.

When administered to ovariectomized rats, an extract of *S. williamsii* increased serum calcium levels, with a concomitant decrease in urinary calcium excretion [334]. It also suppressed the elevated serum alkaline phosphatase and osteocalcin levels as well as the urinary deoxypyridinoline level in ovariectomized rats, in addition to improvement of the biomechanical strength of cortical bone and trabecular bone mass [334]. In a similar experiment using mice, the plant extract increased tibial bone mineral density and exerted beneficial effects on the microarchitecture of trabecular bone [335]. It suppressed the elevated Cbfa1 and cathepsin K mRNA levels and enhanced the OPG/RANKL mRNA expression ratios in the tibia [335].

In vitro results are also available to demonstrate the osteoprotective activity of *S. williamsii*. In UMR-106 osteoblast-like cells, the plant extract was found to increase the osteoprotegrin/RANKL mRNA ratio, in favor of suppressing osteoclastogenesis [334]. In vitro study also showed that the extract reduced the number of TRAP-positive cells in RANKL-induced RAW264.7 cells [336]. An active fraction obtained from elution by aqueous ethanol over D101 macroporous resin contained lignans and phenolic acids. [336,337]. Subsequent studies led to the identification of (7*R*,8*S*)-ficusal (**111**, Figure 2), (7*R*,8*S*)-ceplignan (**112**, Figure 2), (7*R*,8*S*)-dehydrodiconiferyl alcohol (**113**, Figure 2), (7*R*,8*S*)-dehydrodiconiferyl alcohol-γ’-methyl ether (**114**, Figure 2), and samwinol (**115**, Figure 2) as active principles to promote proliferation of the UMR-106 cells [338]. A lignan, namely, (+)-erythro-1-(4-hydroxy-3-methoxyphenyl)-2-[4-(3-hydroxypropanyl)-2-methoxyphenoxy]-1,3-propanediol (**116**, Figure 2) was reported to induce mRNA expressions of Runx2, alkaline phosphatase and osteocalcin, and increase the OPG/RANKL ratio; it failed to bind to either ERα or ERβ and did not activate ERE-luciferase activity via ER. The compound, however, induced phosphorylation of ERK as well as the phosphorylation of ERα at serine-118. These finding suggested the involvement of a ligand-independent, ERE-independent, and MAPK-mediated rapid nongenomic estrogen receptor signaling pathway [339].

Apart from the lignans, vanillic acid (**117**, Figure 4) obtained from this plant was claimed to be responsible for the bone protective effect through the MAP kinase (MEK/ERK)-mediated estrogen receptor signaling pathway [340].

### 3.19. Sophorae Fructus (Fruit of Sophora japonica L. [syn. Styphnolobium japonicum (L.) Schott.])

Sophorae Fructus, derived from the fruits of *Sophora japonica*, is an herbal medicine to “expel excessive heat” from the body and to “cool the blood”.

An isoflavone glycoside-rich extract of Sophorae Fructus was reported to up-regulate the growth factors IGF-1 and TGF-β in rat bone marrow cells [341]. Genistein [342,343] and 8-prenylkaempferol (**118**, Diagram 1) [344] isolated from the plant were shown to be active in promoting the differentiation and maturation of osteoblasts. In particular, the latter was found to accelerate osteoblast maturation through the bone morphogenetic protein-2/-38 pathway and activation of Runx2 transcription [344].

Apart from the fruit part, the seeds of *Sophora japonica* were found to contain estrogenic ingredients, of which sophoricoside (**119**, Figure 6) displayed anti-osteoporotic activity in ovariectomized rats [345]. From the root of *S. flavescens*, on the other hand, (2*S*)-2’-methoxykurarinone (**120**, Figure 5) was shown to inhibit osteoclast differentiation through the down-regulation of the RANKL-induced MAPKs and c-Fos-NHATc1 signaling pathways [346].

### 3.20. Visci Herba [Twig of Viscum coloratum (Kom.) Nakai]

Visci Herba is the twigs of *Viscum coloratum*. The herbal drug is known for its medicinal effects for treating arthritic pain and joint weakness.

A study has shown the inhibitory activity of an ethyl acetate fraction of *Viscum coloratum* on the formation of osteoclast-like cells from mouse bone marrow cells [347]. In ovariectomized rats, the extract displayed anti-osteoporotic activity as shown by increased bone mineral density, bone mineral content, cortical bone thickness, and the X-axis strength index of tibiae. The following active principles were identified: (+)-syringaresinol *O*-β-glucopyranoside (**121**, Figure 2), 2-homoeriodictyol 7-*O*-β-glucopyranoside (**122**, Figure 5), and viscumneoside I (**123**, Figure 5) [347]. Four flavonoids, namely, 2-homoeriodictyol 7-*O*-β-glucopyranoside and viscumneoside I, viscumneoside IX (**124**, Figure 5) and viscumneoside X (**125**, Figure 5), were also reported to inhibit the formation of osteoclast-like multinuclear cells in mouse calvarial osteoblasts [348].

### 3.21. Yams (Dioscorea spp.)

Many species of the *Dioscorea* genus are known as yams and they possess various medicinal properties, including *in vitro* anti-osteoporotic potentials, such as *D. spongiosa* J.Q. Xi. M. Mizuno & W.L. Zhao [349], *D. alata* L. [350], and *D. batatas Decne*. [351].

Diosgenin (**126**, Figure 4), a common steroidal saponin found in yam, could enhance the proliferation of MC3T3-E1 cells with up-regulation of bone marker expressions such as Runx2 and osteopontin [352].

Diarylheptanoids and lignans were obtained from *D. spongiosa* and some of them displayed inhibitory activity against bone resorption in the parathyroid hormone-treated parietal bone of mice. The active compounds included diospongin B (**127**, Figure 4), diospongin C (**128**, Figure 4), piperitol (**129**, Figure 4), sesaminone (**130**, Figure 2), and syrinaresinol (**131**, Figure 2) [353]. From the same plant species, a series of triterpene glycosides were demonstrated to exhibit stimulatory activities on proliferation and/or mineralization, inhibitory activity on bone resorption and/or formation [354].

### 3.22. Miscellaneous Compounds

Formononetin (**132**, Figure 6) is present in *Sophora flavescens* Aiton [355], *Astragalus mongholicus* Bunge and *Trifolium pretense* L. [356] and it has been shown to prevent ovariectomy-induced bone loss in rats by increasing trabecular bone areas within the tibia and lumbar vertebrae [357]. It also up-regulated BMP-2 expression in a high-throughput assay using MC3T3-E1 cells transfected with mouse BMP-2 promoter-luciferase [355]. In vitro osteoblastic activity was shown to be associated with the p38 MAPK pathway, without observable effects on estrogen receptor and osteoclast differentiation [358]. In female rats, the compound increased bone mineral density [358]; and in ovariectomized rats, it enhanced bone biomechanical properties (maximum load and fracture load) and improved the chemical composition of bone (water content and mineral content) [359].

Isoformononetin (**133**, Figure 6) is an isoflavone found in *Pueraria lobate* [360] as well as other Chinese medicinal herbs such as *Ormosia henryi* Prain [361] and *Oxytropis falcate* Bunge [362]. It exhibited both anti-apoptotic and differentiation-promoting activities on osteoblasts that might involve the activation of MEK/ERK and Akt pathways [266]. In ovariectomized osteopenic rats, isoformononetin treatment restored trabecular microarchitecture, increased new bone formation, increased the serum osteogenic marker (procollagen N-terminal propeptide), decreased resorptive marker (urinary C-terminal teleopeptide of type 1 collagen) and diminished osteoblast apoptosis in bone [363].

The polysaccharides of *Lycium babarum* L. has been shown to protect against dexamethasone-induced osteoporosis in rats by improved bone mineral density, serum alkaline phosphatase activity, and calcium and phosphorus contents. The urinary calcium/creatinine and phosphorus/creatinine ratios were lowered after treatment with *Lycium* polysaccharides [364]. The root extract also promoted the proliferation and differentiation of C3H10T1/2 and MC3T3-E1 cells, as well as elevating the bone mineral density in ovariectomized mice [365].

From the root of *Paeonia lactiflora* Pall., 6’-*O*-β-d-glucopyranosylalbiflorin (**134**, Figure 7) was obtained and shown to be able to increase the alkaline phosphatase activity and nodule mineralization of MC3T3-E1 cells [366].

Ferutinin (**135**, Figure 7) is a sesquiterpene found in *Ferula* spp. and a phytoestrogen acting as an agonist of estrogen receptor α [367,368,369]. It was shown to restore histomorphometrical damages in ovariectomized rats by improving the trabecular and cortical bone from lumbar vertebrae and femur [370,371]. Inhibition of bone resorption was suggested [372]. While ferutinin acted similarly to estradiol benzoate on the uterus stimulating endometrial and myometrial hypertrophy, it increased apoptosis in uterine luminal and glandular epithelia, suggesting a protective function against uterine carcinoma [373]. Similar protection in the mammary gland was also observed [374]. In an *in vitro* study using stem cells derived from human amniotic fluid and from the dental pulp, ferutinin promoted the expressions of osteocalcin, osteopontin, collagen I, Runx2 and osterix; it also increased calcium deposition and osteocalcin secretion in the culture medium [375]. In a rat model of cranial defects treated with implanted scaffold containing amniotic fluid stem cells and collagen, oral administration of ferutinin resulted in better improvement of bone regeneration as shown by histomorphometric, immunohistochemical and immunofluorescence analyses [376].

Resveratrol (**136**, Figure 4) is a stilbene polyphenolic present in many plant species such as grapes, mulberries and medicinal herbs such as *Polygonum cuspidatum* Siebold & Zucc. (syn. *Reynoutria japonica* Houtt.). It has estrogenic, anti-inflammatory, antioxidant and proliferative properties [377], and potential applications to improve bone health have been suggested [378,379]. In early studies, resveratrol was found to stimulate the proliferation and differentiation of MC3T3-E1 [380] and human bone marrow mesenchymal stem cells [381]. Mechanistic studies have subsequently demonstrated the involvement of an estrogen receptor-dependent mechanism coupling to ERK1/2 activation [381], the Wnt signaling pathway [382], an upregulation of Runx2 gene expression via the SIRT1/FOXO3A axis [383,384]. Apart from its effects on osteoblastogenesis, resveratrol prevented RANKL-induced osteoclast differentiation, likely through inhibition of reactive oxygen species (ROS) production and/or deacetylation of RANKL-induced NF-κB and inhibition of NF-κB transcriptional activation [385,386]. Resveratrol has been reported to modulate biomarkers of bone metabolism in animal studies as well [387]. It increased epiphysial bone mineral density and inhibited the decrease of femur bone calcium content in ovariectomized rats [388]. Improvements in bone mineral density and trabecular microarchitecture were observed without hyperplastic effects on the uteri of ovariectomized rats [389]. In a hind-limb immobilization (tail-suspension) rat model, feeding of resveratrol for 45 days could prevent against bone loss as demonstrated by an increase in tibial and femoral bone mineral density and preservation of trabecular bone in the proximal tibial metaphysis [390]. However, contradictory results were observed in another study of 21-day treatment [391]. In spinal cord-injured rats, treatment with resveratrol attenuated sublesional bone loss (as demonstrated by bone mineral density, bone mineral content, bone structure and mechanical strength); and the effects were associated with abating oxidative stress, attenuating inflammation, depressing PPARγ signaling, and restoring Wnt/β-catenin and IGF-1 signaling [392]. Resveratrol was found to improve the repair of calvarial bone defects and the biomechanical retention of titanium implants in the tibia of rats [393]. In the same model, the compound up-regulated the gene expressions of osteogenic markers such as BMP-2, BMP-7 and osteopontin [393]. Using aged (22-month) male rats, feeding with resveratrol resulted in improved bone microstructure (higher bone volume, bone trabecular number, and cortical thickness and lower spacing between trabeculae) and biomechanical properties (higher flexural modulus, stiffness, and ultimate load), suggesting it might be useful as anti-aging therapy to resist aged-induced bone loss [394]. Similar results were observed after resveratrol treatment in 33-month-old male rats that were hind-limb-suspended or kept ambulatory [395]. In a study of osteogenic biomaterials, resveratrol was incorporated in scaffold containing porous poly-ε-caprolactone grafted with acrylic acid and subjected to *in vitro* and *in vivo* studies. The osteogenic effect of this scaffold was demonstrated by increased alkaline phosphatase activity and enhanced mineralization in rat bone marrow stromal cells; and in a rat calvarial defect model, the implant enhanced the formation of bone-like structures that were positively immunostained for bone sialoprotein [396].

Ginsenosides are triterpene glycosides in ginseng and related *Panax* species. Early studies have reported the osteoprotective effects of ginsenosides [397,398]. More recently, several ginsenosides were demonstrated to possess bone anabolic and/or anti-resorption activities. Thus, ginsenoside Rb1 (**137**, Figure 3) inhibited RANKL-induced osteoclast differentiation and TNFα mRNA expression in RAW 264.7 cells. It was further shown to inhibit the JNK and p38 MAPKs pathways, and consequently down-regulating the gene expression of c-Fos and NFATc1 [399]. On the other hand, in a hydrogen peroxide-damaged MC3T3-E1 cell model, ginsenoside Rb2 (**138**, Figure 3) was able to promote cell proliferation, increase alkaline phosphatase activity, elevate calcium mineralization and mRNA expressions of osteocalcin (OCN) and osteopontin (OPN). At the same time, it suppressed the expressions of RANKL and IL-6, and inhibited the production of reactive oxygen species (ROS) [400]. The compound also protected dexamethasone-induced apoptosis in primary murine bone marrow mesenchymal stem cells, likely by inducing the Ras-ERK1/2 signaling pathway through the GPR120 receptor [401]. In ovariectomized mice, ginsenoside Rb2 reduced oxidative stress and improved the microarchitecture of trabecular bones and increased bone mineral density of the fourth lumbar vertebrae and the distal femur [400]. Interestingly, ginsenoside Rd (**139**, Figure 3) was reported to possess stimulatory activity on osteoblastic differentiation and mineralization in MC3T3-E1 cells as shown by increased levels of BMP-2, phosphorylated AMP-activated protein kinase (pAMPK), and Smad1/5 [402]. Ginsenoside Rg1 (**140**, Figure 3) has been reported to stimulate osteoblast proliferation and increase alkaline phosphatase activity [398]; it also enhanced the proliferation and osteogenic differentiation of human periodontal ligament stem cells and human dental pulp stem cells, up-regulating the expressions of alkaline phosphatase, OCN, BMP-2, FGF2 [403,404]. Nevertheless, the osteoprotective effect of ginsenoside Rg1 could not be demonstrated in ovariectomized mice [405]. On the other hand, ginsenoside Rg3 (**141**, Figure 3) was reported to display inhibitory activity against osteoclastogenesis in RAW 264.7 cells, as evidenced by reduction of mRNA expressions of markers such as RANK, TRAP, and cathepsin K through the down-regulation of the p38 and JNK pathways [406]. In a sample of fermented red ginseng root, in which the contents of ginsenoside Rg3 was significantly enriched, the differentiation and mineralization in MC3T3-E1 cells was found to be enhanced (compared to non-fermented sample) [407]. A mixture of ginsenosides Rg5 (**142**, Figure 3) and Rk1 (**143**, Figure 3) was shown to enhance the osteoblastic function of MC3T3-E1 cells by displaying increased alkaline phosphatase activity and type 1 collagen contents, as well as up-regulation of mRNA expressions of BMP-2 and Runx2 [408]. Ginsenoside Rh1 (**144**, Figure 3) shared the same properties [409]. On the other hand, ginsenoside Rh2 (**145**, Figure 3) demonstrated a suppression of RANKL-induced osteoclastogenesis both *in vitro* and *in vivo* through down-regulation of NF-κB, NFATc1 and c-Fos [410]. Interestingly, the anti-osteoclastic activity seems to be stereochemically specific to the 20(*R*) isomer when evaluated in RAW 264 cells [411]. The 20(*S*)-ginsenoside Rh2 was shown to enhance differentiation and mineralization of osteoblastic MC3T3-E1 cells through the protein kinase D (PKD) and AMP-activated protein kinase (AMPK) signaling pathways [412,413].

## 4. Concluding Remarks

The current knowledge of natural products suggests that they are a viable source of potential osteoprotective agents. There have been many biological and pharmacological studies, both *in vitro* and *in vivo*, demonstrating that a wide variety of natural products possess potential beneficial effects on maintaining or promoting bone health. Table 2 summarizes the Chinese herbal medicine sources of these bioactive molecules. These substances may be useful as alterative medicines for osteoporosis, especially as preventive agents or as treatment at the early stages, along with exercise and calcium/vitamin D supplementation, to slow down bone loss. Nevertheless, clinical data are nonexistent, except for limited reports on the phytoestrogens derived from soy and red clover as well as a study on the *Epimedium* flavonoids. The efficacy and safety of most, if not all, of these natural compounds is unproven.

This review clearly shows the osteoprotective potential of natural products, but it may leave the readers with a series of questions. Do these natural products act on similar biological targets? Do they work on multiple targets? Why can compounds with diverse chemical structures share similar effects on osteoblasts/osteoclasts? Clearly an essential area that requires better understanding is the mechanism of action. Without probing into where and how the molecules interact with the cellular components, it is difficult to fully appreciate the therapeutic potential of these substances. Another area that requires further attention is the dosage which has not been covered in the present review. The current literature indicates that many studies do not include dose-response relationships, which makes it difficult to extrapolate into human situations. In addition, comparison of study results is not easy, particularly those obtained in different laboratories, due to variations in experimental conditions.

There seems to be no clear answers to many questions at this time. Certainly further work is warranted to identify the mechanisms of action, to optimize the activity (e.g., by way of structural modification), to ensure safety, and ultimately, to confirm clinical results.

## Figures and Tables

**Table 1:** Commonly used biomarkers of bone turnover.

Bone Formation	Bone Resorption
**Osteoblast enzyme**	**Osteoclast enzyme**
Total alkaline phosphatase [52]	Tartrate-resistant acid phosphatase (TRAP) [54]
Bone-specific alkaline phosphatase [53]	Cathepsin K [55]
**Matrix protein**	**Collagen degradation product**
Osteocalcin [56,57]	Hydroxyproline [57]
	Pyridinoline [58]
	Deoxypyridinoline [58]
**Product of collagen synthesis**	**Cross-linked telopeptide of type I collagen**
Procollagen type I C-terminal extension peptide (P1CP) [59]	N-telopeptide of type I collagen [59]
Procollagen type I N-terminal extension peptide (P1NP) [59]	C-telopeptide of type I collagen [59]
	C-telopeptide generated by matrix metalloprotieinases [59]
	**Others**
	Osteopontin (OPN) [60]
	Receptor activator of nuclear factor Kappa B ligand (RANKL) [61]
	Osteoprotegerin (OPG) [62]

**Table 2:** Botanical sources of natural products from Chinese medicinal herbs showing osteoprotective potential.

Latin Name	Chinese Name	Plant Part	Major Active Molecule
*Actaea heracleifolia* (Kom.) J. Compton; *A. dahurica* (Turcz. ex Fisch. & C.A. Mey.) Franch.; *A. foetida* L.	Sheng-Ma	Rhizome	cimicidol-3-*O*-β-xyloside, cimicidanol-3-*O*-β-xyloside, acetylacteol-3-*O*-arabinoside [73]
*Astragalus mongholicus* Bunge	Huang-Qi	Root	formononetin [355,356,357,358,359]
*Carthamus tinctorius* L.	Hong-Hua	Flower	matairesinol, tilianine, acacetin and their derivatives [72]
*Cistanche deserticola* Y.C. Ma; *C. tubulosa* (Forssk.) Beck	Rou-Cong-Rong	Stem	8-hydroxy-2,6-dimethyl-2-octenoic acid [81], echinacoside [85,86,87]
*Cordyceps sinensis* (Berk.) Sacc.	Dong-Cong-Xia-Cao		cordycepin [94,95,96,97]
*Dioscorea* spp.		Rhizome	diosgenin [353], diospongins B and C, piperitol, sesqminone, syrinaresinol [353]
*Dipsacus asper* Wall. ex C.B. Clarke; *D. japonicas* Miq.	Xu-Duan	Root	asperosaponins V and VI [103,106], hederagenin-3-*O*-(2-*O*-acetyl)-α-l-arabinopyranoside [104]
*Drynaria fortunei* (Kunze ex Mett.) J. Sm.	Gu-Sui-Bu	Rhizome	naringin and other flavos [116,117,118,119,120,121,122,123,124,125,126,127,128,129,130,131,132,133,134,135,136,137,138,139,140,141,142,143,144,145,146,147]
*Eclipta prostrata* L.	Mo-Han-Lian	Above-ground parts	diosmetin, 3′-hydroxybiochanin A, 3′-*O*-methylorobol [150], echinocystic acid [156], wedelolactone [157]
*Epimedium brevicornum* Maxim.; *E. sagittatum* (Siebold & Zucc.) Maxim.; *E. pubescens* Maxim.; *E. koreanum* Nakai	Yin-Yang-Huo	Leaf	icariin [158,159,160,161,162,163,164,165,166,167,168,169,170,171,172,173,174,175,176,177,178,179,180,181,182,183,184,185,186,187,188,189,190,191,192,193,194,195,196,197,198,199,200,201,202,203,204,205,206,207,208,209,210], epimedins A, B and C [178,211], baohuoside-1 [189,212,213], maohuoside A [214,215], sagittatoside A [189], ikarisoside A [216], icaritin [209,220,221,222,223,224,225,226,227,228,229,230], icariside I and II [209,219]
*Erythina variegate* L.	Hai-Tong-Pi	Bark	6-prenylgenistein, 8-prenylgenistein, 6,8-diprenylgenistein [233]
*Eucommia ulmoides* Oliv.	Du-Zhong	Stem bark	geniposidic acid, geniposide, aucubin [237,238], 5-hydroxymethyl-2-furaldehyde [239]
*Ferula* spp.	A-Wei	Resin	Ferutinin [370,371,372,373,374,375,376]
*Ligustrum lucidum* W.T. Aiton	Lu-Zhen-Zhi	Fruit	oleanolic acid [244,245,246,247,248,249,250], ursolic acid [250,251,252,253], tyrosol, hydroxytyrosol, oleuropein, and others [256]
*Lycium babarum* L.	Gou-Qi-zi	Fruit	polysaccharide [364]
*Morinda officinalis* F.C. How	Ba-Ji-tian	Root	physicion, rubiadin, rubiadin-1-methyl ether, 2-hydroxy-1-methoxyanthraquinone, 1,2-dihydroxy-3-methylanthraquinone, 1,3,8-trihydroxy-2-methoxyanthraquinone, 2-hydroxymethyl-3-hydroxyanthraquinone, 2-methoxyanthraquinone, scopoletin [261,262]
*Ormosia henryi* Prain	Lu-Mu	Root	isoformononetin [266,363]
*Oxytropis falcata* Bunge	Lian-Xing-Ji-Dou	Whole plant	isoformononetin [266,363]
*Paeonia lactiflora* Pall.	Bai-Shao	Root	6′-*O*-β-d-glucopyranosylalbiflorin [366]
*Panax* spp.	Ren-Shen	Root	ginsenosides [397,398,399,400,401,402,403,404,405,406,407,408,409,410,411,412,413]
*Podocarpium podocarpum* (DC.) Yang et Huang		Whole plant	podocarnone, luteolin, astragalin, afzelin, kaempferitrin, rutin, quercetin-7-*O*-d-glucopyranoside, genistein, laburnetin, wighteone, luteone, 7-*O*-methyl-luteone [265], cajanin [266]
*Polygonum cuspidatum* Siebold & Zucc.	Hu-Zhang	Root and Rhizome	resveratrol [378,379,380,381,382,383,384,385,386,387,388,389,390,391,392,393,394,395,396]
*Psoralea corylifolia* L.	Bu-Gu-Zhi	Fruit	psoralen [274,275,276], isopsoralen [278,279], psoralidin [280], corylin [281], bavachin, isobavachin [282,283], neobavaisoflavone [284], bakuchiol [283,285], bavachalcone [286]
*Pueraria lobate* (Willd.) Ohwi	Ge-Gen	Root	isoformononetin [266,363], puerarin and its 6′′-xyloside [297,298,299,300,301,302,303,304,305,306,307,308,309,310,311]
*Rehmannia glutinosa* (Gaertn.) DC.	Di-Huang	Root	acteoside [314,315,316]
*Salvia miltiorrhiza* Bunge	San-Shen	Root/rhizome	salvianolic acids A and B [322,323], tanshinones I, IIA and VI, cryptotanshinone, 15,16-dihydrotanshinone I [324,325,326,327,328,329,330,331]
*Sambucus williamsii* Hance	Jie-Gu-Mu	Stem	ficusal, ceplignan, dehydrodiconiferyl alcohol, dehydrodiconiferyl alcohol-γ′-methyl ether, samwinol [338], erythro-1-(4-hydroxy-3-methoxyphenyl)-2-[4-(3-hydroxypropanyl) -2-methoxyphenoxy]-1,3-propanediol [339], vanillic acid [340]
*Sophora japonica* L.	Huai-Jiao	Fruit, seed and root	genistein [342,343], 8-prenylkaempferol [344], sophoricoside [345], 2′-methoxykurarinone [346]
*Sophora flavescens* Aiton	Ku-Shen	Root	formononetin [355,356,357,358,359]
*Trifolium pretense* L.	Hong-Che-Hou-Cao	Inflorescence and twig	formononetin [355,356,357,358,359]
*Viscum coloratum* (Kom.) Nakai	Hu-Ji-Sheng	Twig	syringareninol *O*-β-glucopyranoside, 2-homoeriodictyol 7-*O*-β-glucopyranoside, viscumneosides I, IX and X [347,348]
